# Distinct cell type–specific mechanisms underlie cognitive dysfunction during persistent integrated stress response activation

**DOI:** 10.1073/pnas.2537017123

**Published:** 2026-07-14

**Authors:** Kristof Torkenczy, Lucas C. Reineke, Sean W. Dooling, Benjamin W. Henderson, Benjamin Yang, Dongze He, Richard M. Myers, Peter Walter, Stefka Tyanova, Mauro Costa-Mattioli

**Affiliations:** ^a^ Altos Labs, Inc., Bay Area Institute, Redwood City, CA 94665; ^b^ HudsonAlpha Institute for Biotechnology, Huntsville, AL 35806; ^c^ Department of Neuroscience, Baylor College of Medicine, Houston, TX 77030

**Keywords:** cognitive decline, single-cell RNA-sequencing, single-cell ATAC-sequencing, cellular homoeostasis

## Abstract

Persistent activation of the integrated stress response (ISR) is a central driver of cognitive decline in both neurodevelopmental and neurodegenerative disorders. However, the cell type–specific mechanisms underlying these deficits remain poorly understood. By integrating single-cell RNA-seq and single-cell assay for transposase-accessible chromatin sequencing, we generated a brain ISR atlas using *Ppp1r15b*^R658C^ mice, a clinically relevant model of intellectual disability characterized by selective and persistent ISR activation. We find that distinct brain cell types differentially engage transcriptional and chromatin remodeling programs. Notably, selective deletion of the major ISR downstream effector ATF4 in GABAergic neurons, but not in glutamatergic neurons, exacerbates ISR-mediated cognitive decline in *Ppp1r15b*^R658C^ mice, demonstrating that different neuronal subtypes rely on distinct ISR effectors. We define a molecular single-cell signature of persistent ISR activation that serves as a metric of ISR-mediated cellular vulnerability and as a biomarker for cognitive dysfunction across human cognitive disorders. These findings demonstrate that cell type–specific responses drive cognitive dysfunction during persistent ISR activation.

## Significance

Persistent activation of the integrated stress response (ISR) is causally linked to cognitive dysfunction across neurological disorders, yet specific cell-type vulnerabilities remain unknown. Using single-cell RNA and ATAC-seq in a recently developed intellectual disability model caused by an activating human variant in a central ISR component, we show that distinct gene regulatory programs are engaged across neuronal populations. Molecular experiments reveal that the ISR effector ATF4 protects GABAergic neurons during ISR-induced impairment, whereas its deletion in glutamatergic neurons has no effect. We further define a molecular signature of persistent ISR activation that serves as a metric for cell-type susceptibility and a biomarker for neurological disorders. These findings identify potential therapeutic avenues to reverse cognitive dysfunction by targeting cell-specific stress pathways.

The integrated stress response (ISR) is a conserved signaling network that restores cellular homeostasis by regulating protein synthesis (1, 2). It is primarily controlled by phosphorylation of the alpha subunit of the eukaryotic translation initiation factor 2 (eIF2) by a family of dedicated stress-sensing kinases: GCN2, PERK, PKR, and HRI (3). Phosphorylation of eIF2 (eIF2-P) inhibits the activity of the guanine nucleotide exchange factor eIF2B, which is essential for recycling eIF2-GDP to eIF2-GTP and formation of the ternary complex required for translation initiation (4). Consequently, ISR activation reduces global protein synthesis while selectively enhancing the translation of specific mRNAs, such as ATF4, which orchestrates stress adaptation programs. Termination of the ISR and restoration of normal translation are mediated by two eIF2 phosphatase complexes that dephosphorylate eIF2-P (5, 6): the constitutive PPP1R15B•PP1 complex and the inducible PPP1R15A•PP1 complex, the latter being upregulated during acute ISR activation to restore homeostasis.

In the brain, the ISR has evolved beyond its canonical role to dynamically regulate synaptic plasticity, cognitive function and brain health (2, 7). ISR activity modulates both long-term potentiation and depression of synaptic strength, as well as structural plasticity (7–17); cellular processes underlying long-term memory formation (18, 19). Genetic and pharmacological evidence consistently demonstrate that ISR activation impairs long-term memory, whereas inhibition enhances it in both physiological and disease models, including Down syndrome, Alzheimer’s disease, prion’s disease, traumatic brain injury, vascular dementia, and age-related cognitive decline (2, 7, 8, 11, 13, 16, 20–76). These findings, independently reproduced across laboratories worldwide, underscore the strength and generalizability of this framework and have also spurred industry efforts aimed at targeting the ISR to promote brain health (38, 49, 68).

Crucially, this maladaptive persistent ISR activation extends to humans: ISR markers are elevated in the brain of individuals with neurological disorders (39, 77–81), and rare genetic variants in central components of the ISR that constitutively activate the pathway are associated with intellectual disability (82–89). Consequently, ISR inhibition has emerged as a promising strategy for reversing cognitive dysfunction and promoting brain health in a wide range of memory disorders resulting from disrupted cellular homeostasis (2). Thus, a single druggable pathway provides a unifying mechanistic framework and therapeutic opportunity for a spectrum of devastating, previously unrelated diseases (90).

Despite its therapeutic potential, the mechanisms by which persistent ISR activation drives long-term memory deficits and brain pathology remain poorly understood. Because cognitive processes depend on the coordinated activity of diverse neuronal and glial populations, determining which brain cell types are most vulnerable to persistent ISR activation—and identifying their downstream effectors—is critical for understanding how dysregulation of cellular homeostasis impairs brain function.

To address these questions, we applied a comprehensive multiomic strategy combining single-cell RNA sequencing (scRNA-seq) and single-cell assay for transposase-accessible chromatin sequencing (scATAC-seq) in a recently generated model of persistent ISR activation (*Ppp1r15b*^R658C^ mice) (91). These mice harbor a human patient-derived genetic variant (R658C) in the phosphatase cofactor PPP1R15B associated with intellectual disability (83–85). In their brains, the variant disrupts the PPP1R15B•PP1 complex, leading to ISR activation (increased eIF2-P levels) (91). Furthermore, because the inducible negative feedback PPP1R15A•PP1 complex fails to engage in the brains of *Ppp1r15b*^R658C^ mice, the ISR remains persistently activated (91). Consequently, *Ppp1r15b*^R658C^ mice exhibit deficits in protein synthesis, long-term synaptic plasticity, and long-term memory, which are fully reversed by ISR inhibition (91). Thus, the cognitive deficits in *Ppp1r15b*^R658C^ mice are driven specifically by persistent ISR activation.

Determining the specific contributions of the ISR to different cell types during cognitive dysfunction has been challenging. For instance, in neurodegenerative conditions, the ISR is coactivated alongside parallel signaling pathways (92–94), including those involved in inflammation and cell death, making it difficult to deconvolute which transcriptional changes are mediated specifically by the ISR. Similarly, traditional chemical inducers like tunicamycin cause supraphysiological activation of all UPR branches in addition to the ISR, failing to accurately reflect chronic disease. In contrast, the *Ppp1r15b^R658C^* mouse model is clinically relevant and offers two distinct advantages: it ensures specificity by activating the ISR without engaging parallel stress pathways, and it models the persistent ISR activation observed across multiple memory disorders (91). Thus, this model provides a platform to dissect the ISR-driven transcriptome and chromatin landscape at single-cell resolution within a clinically relevant context. Unexpectedly, our analyses revealed that persistent ISR activation impacts different brain cell types in distinct ways, challenging the notion of a uniform ISR response across the brain. Moreover, we demonstrated that different cell types rely on unique ISR effectors to regulate long-term memory. Finally, we defined a molecular signature of persistent ISR activation, which serves as a metric for evaluating the intrinsic susceptibility of different cell types to ISR activation and a biomarker of human cognitive impairment across neurodevelopmental and neurodegenerative disorders.

## Results

### Distinct Patterns of ISR Activation across Different Brain Cell Types.

To understand how the ISR modulates different behavioral states, it is important to determine how it impacts different cell types involved in mnemonic processing. While recent advances in high-throughput single-cell sequencing technology have defined several neuronal and glial cell types based on their transcriptional profile (95, 96), the impact of the ISR within each of these cell-type populations remains poorly understood.

**Fig. 1.**
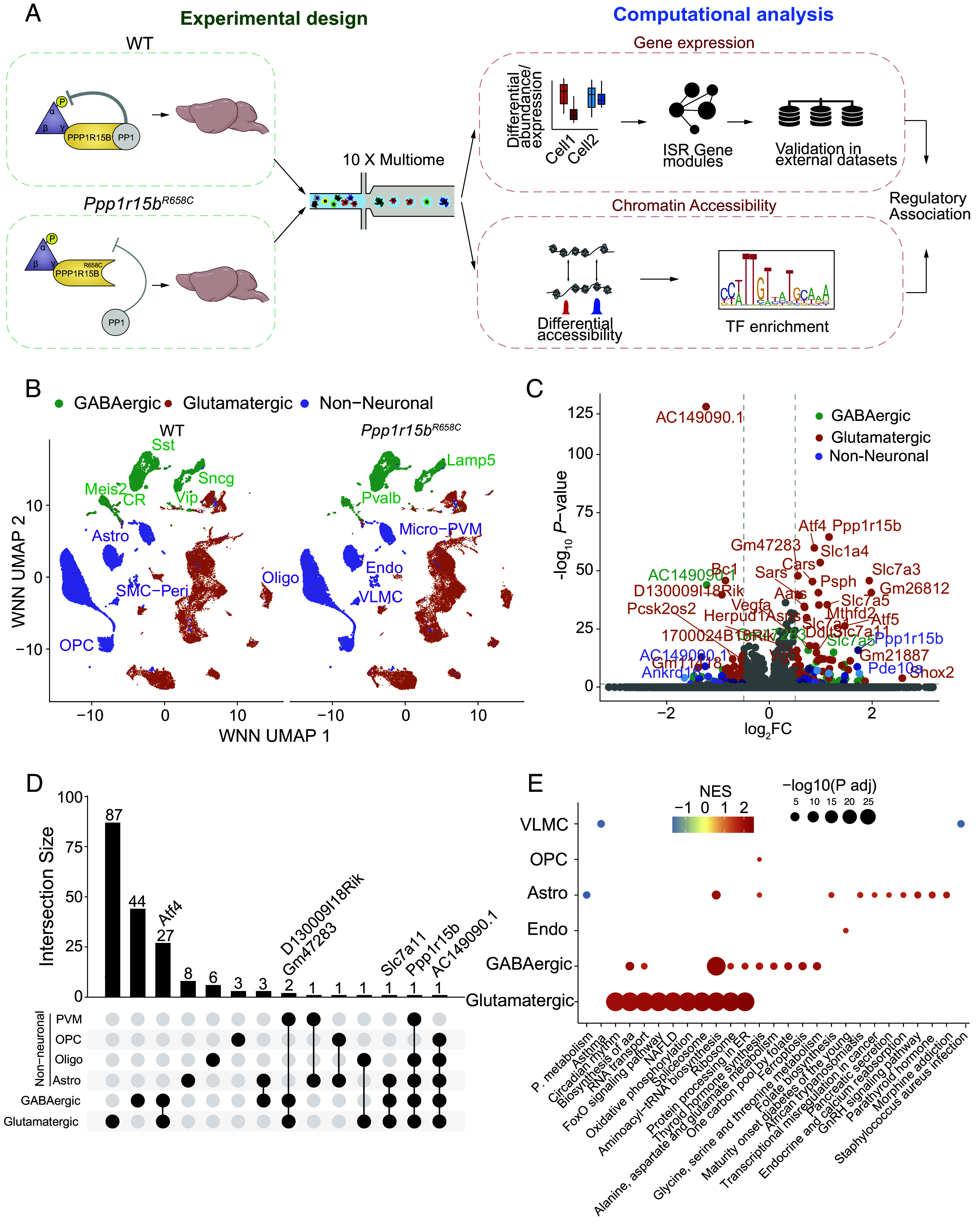
Single-cell multiomic map of persistent ISR activation. (*A*) Overview of the experimental design (*Left*) used to map the single-cell multiomic landscape of brain cell populations in mice with persistent ISR activation (*Ppp1r15b^R658C^*) compared to control (WT) mice (*n* = 7 mice per group). Schematic of the computational analysis pipeline (*Right*) used to identify cell type–specific differences in gene expression and chromatin accessibility between brain samples from WT and *Ppp1r15b^R658C^* mice. TF, Transcription Factor. (*B*) Joint weighted UMAP projections of 85,402 cells from WT (*Left*) and *Ppp1r15b^R658C^* (*Right*) mice, visualized across the three major cell classes: GABAergic, glutamatergic, and nonneuronal lineages. WNN, Weighted Nearest Neighbor. (*C*) Volcano plot of differential gene expression analysis between WT and *Ppp1r15b^R658C^* brain cells. DEGs (dashed lines) with an adjusted *P*-value < 0.05 and a log2-fold change > 0.25 (representing effect size) are categorized by GABAergic, glutamatergic, and nonneuronal lineages. (*D*) Cell type–specific unique and shared DEGs between brain cells from WT and *Ppp1r15b*^R658C^ mice. (*E*) KEGG GSEA of DEGs in each brain cell type in WT and *Ppp1r15b*^R658C^ mice. Significant pathways were selected based on the adjusted *P*-value threshold of <0.05. The normalized enrichment score (NES) and −log10 adjusted *P*-value are represented by the color and size of the dots, respectively.

To address this, we performed single-cell (10×) multiomic sequencing of the cortex from WT and *Ppp1r15b*^R658C^ mice to simultaneously measure gene expression changes (scRNA-seq) and chromatin accessibility (scATAC-seq) (Fig. 1*A* and *SI Appendix*, Table S1). After filtering, we retained 85,402 nuclei covering 28,601 genes and 308,774 *cis*-regulatory elements (CREs). The dataset was robust, showed minimal mitochondrial contamination, high gene detection per cell, strong enrichment at transcription start sites (TSS) and consistent nucleosome signal (*SI Appendix*, Fig. S1 *A* and *B*). Kruskal–Wallis tests confirmed that while quality metrics varied significantly across the large dataset (n = 102,269), the low effect sizes (η^2^ [*H*] ≤ 0.131) indicated high technical consistency between groups.

We performed principal component analysis (PCA) for gene expression and latent semantic analysis (LSI) for chromatin accessibility data (97, 98). Using reference mapping to the Allen Brain Atlas (99) and manual annotation with known cell-type markers, we identified all major brain cell classes. The overall cell-type composition was unchanged between WT and *Ppp1r15b*^R658C^ brains, as determined by weighted-nearest neighbor (WNN) Projection of data using Uniform Manifold Approximation and Projection for Dimension Reduction (UMAP; Fig. 1*B* and *SI Appendix*, Fig. S1 *C* and *D*). Strikingly, ISR-induced genes displayed varying degrees of up-regulation across cell types in *Ppp1r15b*^R658C^ mice (Fig. 1*C*). Despite persistent ISR activation, no gene was consistently regulated across all brain cell types (Fig. 1*D*). One of the broadest changes observed across multiple populations was decreased levels of *AC149090.1*, a phospholipid decarboxylase associated with aging (100) (Fig. 1 *C* and *D* and *SI Appendix*, Fig. S2*A*). In contrast, most of the ISR-responsive genes exhibited cell type–specific expression patterns. For example, while 27 genes (including *Atf4*) were shared between glutamatergic and GABAergic neurons, 87 were unique to glutamatergic neurons, and 44 were specific to GABAergic neurons (Fig. 1*D*). To elucidate the functional consequences of persistent ISR activation, we performed gene set enrichment analysis (GSEA) for each cell type using the KEGG database, ranking genes by the magnitude and significance of their DE. This analysis highlighted the heterogeneous response to persistent ISR activation across brain cells (Fig. 1*E* and *SI Appendix*, Fig. S2 *B* and *C*). Importantly, the differential cell-type responses to persistent ISR activation are unlikely to be attributed to variations in *Ppp1r15b* expression. While we observed statistically significant differences in *Ppp1r15b* transcript levels across cell types (*SI Appendix*, Fig. S3; Kruskal–Wallis *H* test, n = 1,109: χ^2^ (8) = 216.26, *P* < 2.2 × 10^–16^), this variation was not coupled to the magnitude of ISR signature gene expression. Specifically, Spearman correlation analysis across 1,109 metacells revealed no significant relationship between *Ppp1r15b* expression levels and the master ISR regulator *Atf4* (ρ = 0.01, *P* = 0.74), or the downstream gene *Ddit3* (ρ = 0.05, *P* = 0.09; *SI Appendix*, Table S3).

To further assess cell type–specific ISR activity, we quantified the expression of *Atf4*, the gene encoding a key transcription factor (TF) that is a major ISR downstream target (2). While ISR activation triggers *Atf4* mRNA translation (101, 102), transcript levels are also elevated during the response (103, 104). Thus, *Atf4* mRNA levels can serve as a proxy for pathway activation. In the brains of *Ppp1r15b*^R658C^ mice, *Atf4* expression was significantly higher in multiple cell types, including glutamatergic and GABAergic neurons. Oligodendrocytes also displayed elevated *Atf4* expression in the brains of *Ppp1r15b*^R658C^ mice (*SI Appendix*, Fig. S2*A*). Collectively, these findings indicate that persistent ISR drives highly cell type–specific responses in the brain, suggesting that distinct ISR downstream effectors mediate unique functional outcomes across different cell types.

### Identification of an ISR Signature Using scRNA-seq: A Potential Biomarker for Cognitive Disorders and Metric for Cell-Type Susceptibility.

Defining a comprehensive single-cell ISR signature is critical for uncovering cell type–specific vulnerabilities that drive cognitive disorders and for providing precise, clinically relevant biomarkers. The magnitude of the ISR transcriptional response serves as a molecular gauge of cellular susceptibility to homeostatic disruption. Cell types exhibiting a prominent ISR signature represent populations most burdened by stress, necessitating extensive transcriptional reprogramming to restore equilibrium. Conversely, cell types displaying attenuated or negligible ISR activation, despite harboring the same genetic driver, likely possess intrinsic resilience mechanisms, such as superior buffering capacity, enabling them to withstand stress without engaging the canonical response.

While ATF4 target gene expression is often used as a surrogate for ISR activation, ATF4 can be modulated by ISR-independent mechanisms, including mechanistic target of rapamycin (mTORC1)-mediated translation (105–107), promoter methylation, and posttranslational modifications (108). Consequently, relying solely on ATF4 targets risks confounding ISR activity with other signaling pathways. To achieve a more accurate ISR transcriptional readout, a comprehensive ISR signature must therefore extend beyond this canonical target to capture the specific, broad-spectrum regulatory programs that uniquely characterize persistent ISR activation.

**Fig. 2.**
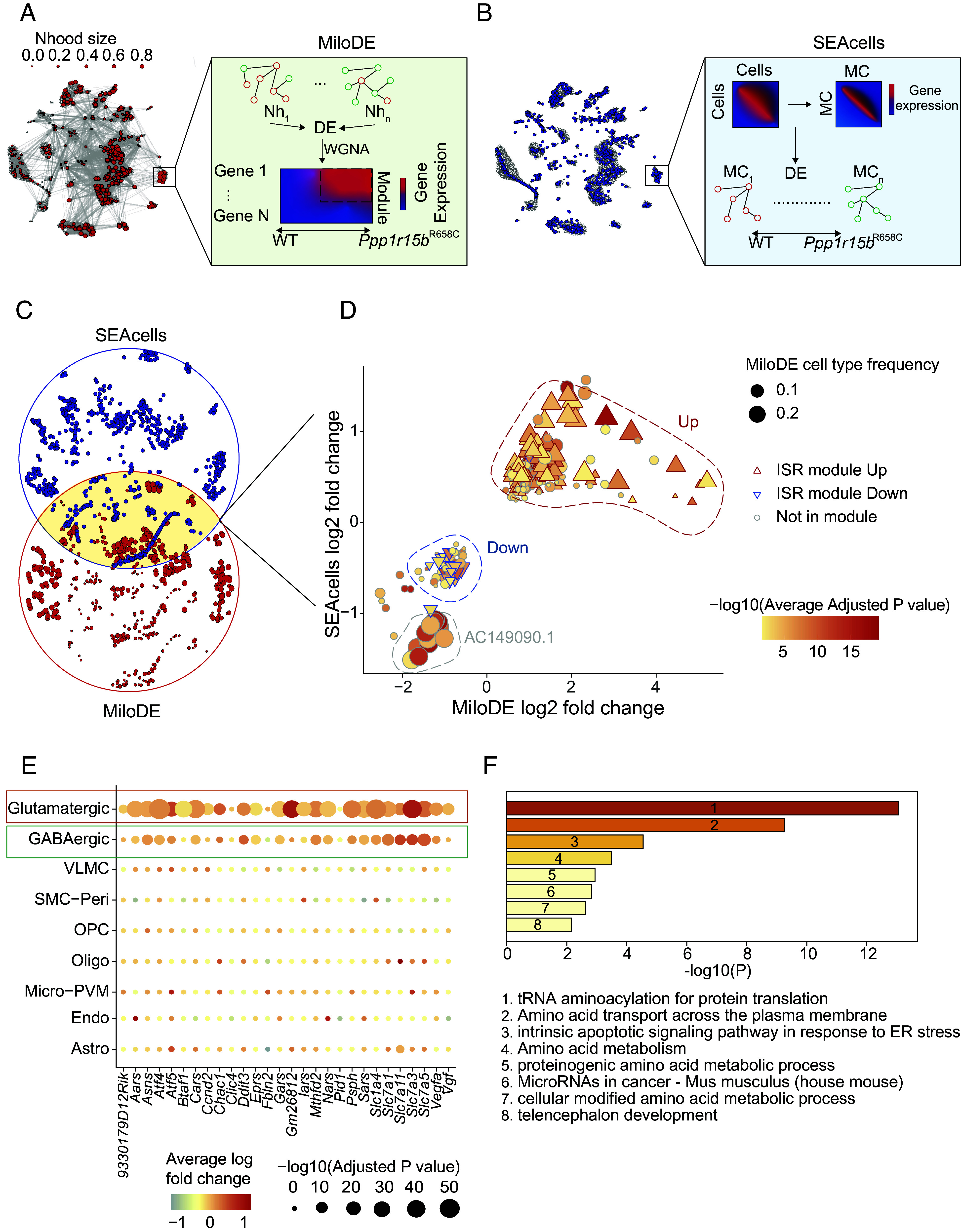
Defining an ISR-specific gene signature. (*A*) Computational workflow for identifying ISR-related gene modules using MiloDE. The *Left* panel shows the identified cell neighborhoods with neighborhood size, and the *Right* panel provides an overview of the approach. (*B*) Computational pipeline for identifying ISR related gene modules using SEAcells. The *Left* panel displays identified metacells, and *Right* panel provides an overview of the approach. MC, Metacell, DE, Differential expression. (*C*) Framework for defining the persistent ISR gene signature. (*D*) Comparison of gene modules derived from MiloDE and SEAcells analyses. DEGs between *Ppp1r15b*^R658C^ and WT mice are categorized into ISR up- and down-modules. Log2-fold change values (representing effect size) for both SEAcells and MiloDE analyses are shown, with adjusted *P*-values indicated. The relative frequency of DEGs is represented by the size of the shapes, with gene membership to the up- and down-modules indicated by shape type. (*E*) Log2-fold change and adjusted *P*-value of the upregulated ISR gene coexpression module identified by SEAcells. (*F*) Metascape analysis of the ISR up-module showing the top 8 enriched biological ontologies.

To this end, we employed metacell-based approaches that cluster similar cells based on gene expression patterns, thereby improving the signal-to-noise ratio in single-cell genomics (109) (*SI Appendix*, Fig. S4). We first used MiloDE (110), a statistical framework designed to detect subtle gene expression changes that are often missed by traditional single-cell methods. This approach identified neighborhoods of cells with significant changes in gene expression across the brains of WT and *Ppp1r15b*^R658C^ mice (Fig. 2*A*). We next applied single-cell weighted gene coexpression network analysis (scWGCNA) to these cell neighborhoods to identify gene modules dysregulated in the brain of *Ppp1r15b*^R658C^ mice. Iterative testing of differential expression (DE) thresholds ensured robustness (*SI Appendix*, Fig. S5). This analysis yielded two highly reproducible gene modules, each containing ~30 genes (*SI Appendix*, Fig. S5 *C* and *D*). The “ISR-up” module showed increased expression of a shared set of ISR genes in several cell types, including glutamatergic and GABAergic neurons, oligodendrocytes, and astrocytes, although the magnitude of the response varied between cell types, and cortical layers (*SI Appendix*, Figs. S5*C* and S6 *A* and *B*). Conversely, the “ISR-down” module consisted primarily of genes reduced in glutamatergic and GABAergic neurons (*SI Appendix*, Figs. S5*D* and S6 *C* and *D* and Table S2). Both modules were generally enriched across cortical layers, while endothelial cells and somatostatin neurons showed minimal DE of these genes (*SI Appendix*, Fig. S6 *B*–*D*). Moreover, we identified a module of upregulated genes only in oligodendrocytes (*SI Appendix*, Fig. S6 *E* and *F*), further supporting the notion that distinct brain cell types respond differently to persistent ISR activation.

To refine the ISR signature, we next employed SEACells (111), which groups single cells with similar expression profiles into high-quality metacells (Fig. 2*B*) to enhance detection of low abundance genes while preserving biological variability. Comparison of differentially expressed genes (DEGs) from SEACells and MiloDE yielded a consensus ISR signature (Fig. 2 *C* and *D*) comprising 28 genes from the ISR-up module (Fig. 2*E*). This ISR signature was generally upregulated across layers of the mouse cerebral cortex in *Ppp1r15b*^R658C^ mice, although some differences in magnitude were observed (*SI Appendix*, Fig. S6*B*). Although a downregulated gene set was also detected (Fig. 2*D*), its mouse-specific nature limited its cross-species utility. Thus, we defined an upregulated gene set as the “ISR signature” for validation. Notably, while approximately two thirds of the signature genes have been previously identified as direct targets of ATF4 (112), the presence of non-ATF4 targets in the ISR signature integrates a broader regulatory input. Gene ontology analysis revealed enrichment in pathways related to tRNA aminoacylation (e.g., *Aars*, *Eprs*, *Nars*, *Sars*), amino acid transport (e.g., *Slc1a4*, *Slc7a11*, *Slc7a3*, *Slc7a5*), and amino acid metabolism (e.g., *Psph*, *Chac1*, *Mthfd2*; Fig. 2*F*), consistent with ISR activation.

**Fig. 3.**
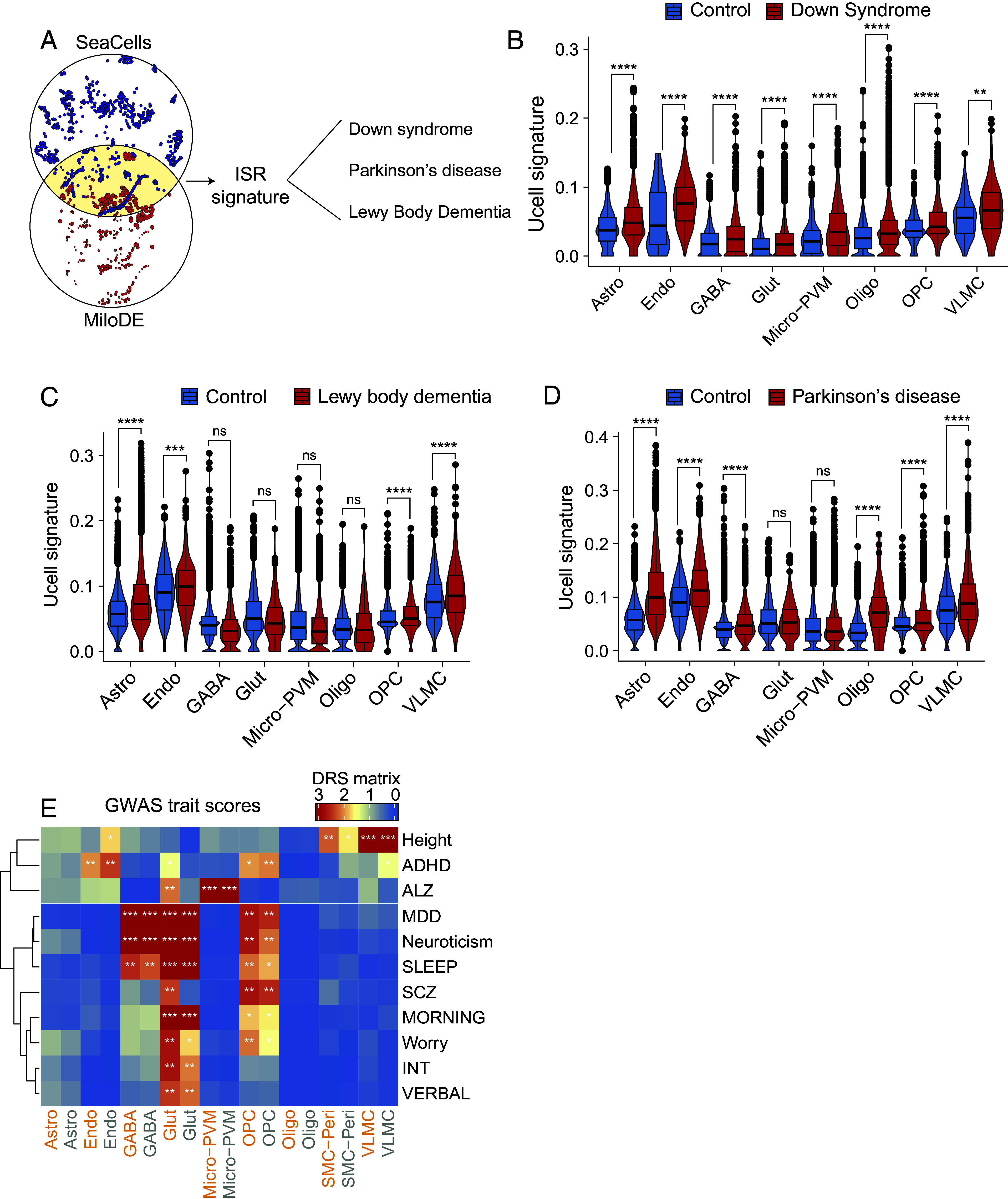
The ISR gene signature is present in human diseases associated with persistent ISR. (*A*) Schematic showing the selection of genes included in the ISR gene signature, and their application to human diseased datasets. (*B*) Ucell scores of the ISR gene signature in control vs. Down Syndrome individuals across cell types (**P < 0.01, ****P < 0.0001). (*C*) Ucell scores of the ISR gene signature in control vs. individuals with Lewy body dementia across cell types (***P < 0.001, ****P < 0.0001). (*D*) Ucell scores of the ISR gene signature in control vs. Parkinson’s disease individuals across cell types (****P < 0.0001). (*E*) Comparison of the ISR gene signature with genes identified in human genome-wide association studies for the indicated diseases. A unified one-sided Monte Carlo test was performed on the data (*P < 0.05, **P < 0.01, ***P < 0.001). DRS, Disease Relevance Score; ADHD, attention deficit/hyperactivity disorder; ALZ, Alzheimer’s disease; MDD, major depressive disorder; Neuroticism; Sleep, sleep deprivation; SCZ, schizophrenia; Morning; Worry; INT, intelligence; Verbal, verbal numeric reasoning.

Given that the ISR is activated in various conditions associated with cognitive dysfunction (2), we next tested whether this signature could serve as a reliable biomarker (Fig. 3*A*). Analysis of human single-cell data from Down Syndrome (113)—a neurodevelopmental disorder (114) where ISR activation is causally linked to the synaptic and cognitive deficits observed in a mouse model of the disease (39)—revealed upregulation of the ISR genes across all cell types (Fig. 3*B* and *SI Appendix*, Fig. S7*A*), demonstrating that the ISR is activated in the brains of individuals with Down syndrome. Similarly, analysis of single-cell datasets from individuals with either Lewy Body Dementia or Parkinson’s disease (115) showed ISR upregulation (Fig. 3 *C* and *D*), although the extent and affected cell types varied between the two diseases. Interestingly, in Parkinson’s disease, the ISR signature was detectable in astrocytes (*SI Appendix*, Fig. S7 *B* and *C*), whereas in both diseases, neurons displayed consistent ISR activation.

Moreover, using single-cell disease relevance scoring (116), which integrates scRNA-seq data with genome-wide association studies, we found the ISR was unrelated to traits such as height and anxiety but showed significant association in glutamatergic neurons in both Alzheimer’s disease and schizophrenia (Fig. 3*E*). While ISR activation is known to contribute to the cognitive decline in Alzheimer’s disease (48, 117, 118), its potential involvement in schizophrenia is an interesting hypothesis that requires further testing.

### Cell Type–Specific Roles of ISR Downstream Effectors during Persistent ISR Activation.

**Fig. 4.**
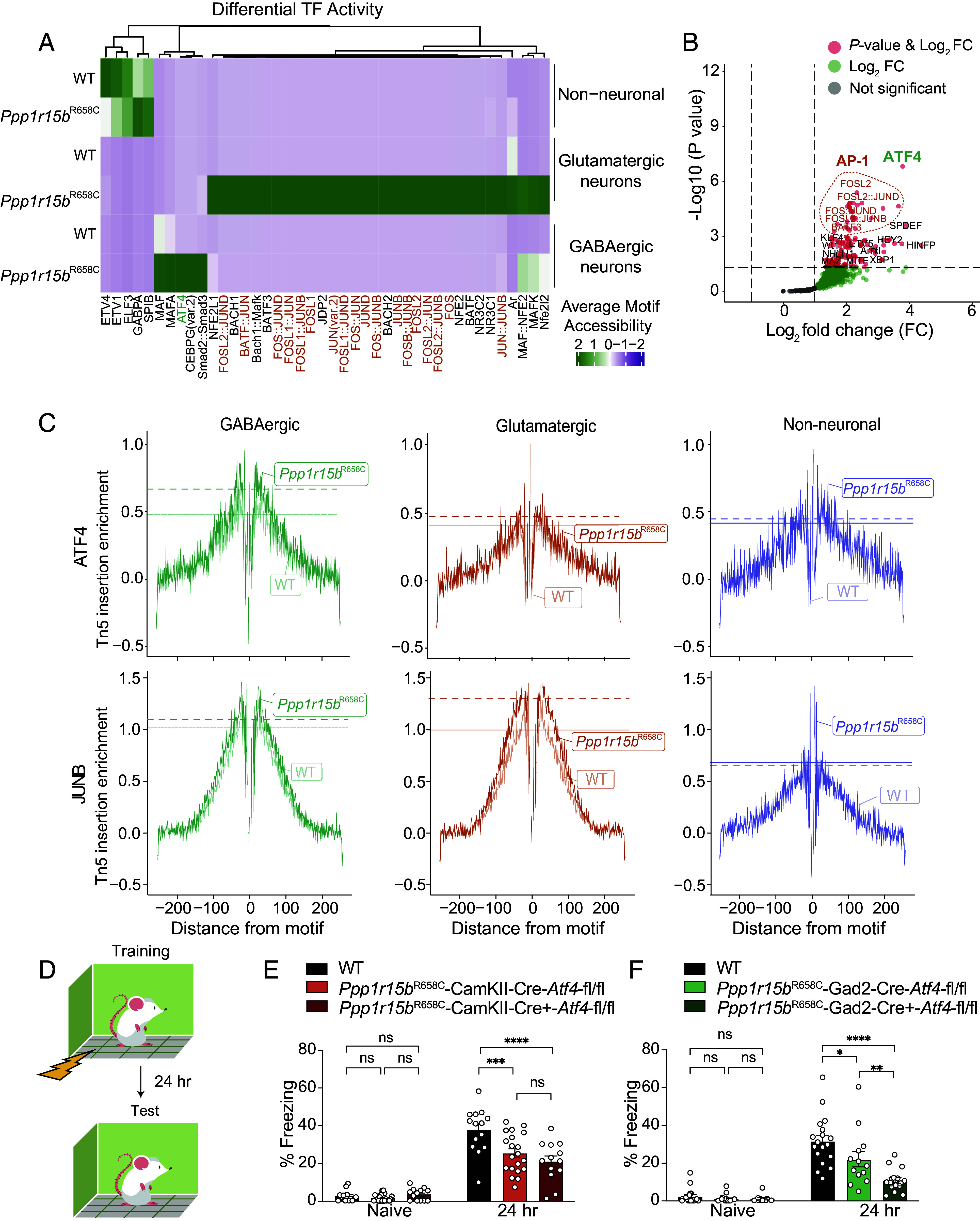
Epigenetic characterization of cell type–specific ISR regulation. (*A*) TF activity across cell types and conditions, shown as ChromVar deviation Z-scores. ATF4 and AP-1 family members are highlighted in green and orange, respectively. (*B*) Volcano plot of top TF motif enrichment in ATAC-seq peaks associated with gene expression changes (*P*-value < 0.001 and z-score > 0), using a GC-matched hypergeometric background. Members of the AP-1 family of TFs are annotated and outlined in brown. (*C*) Bias corrected ATAC-seq footprinting signal centered around ATF4 (*Top*) and JUNB (*Bottom*) motifs, aggregated from peaks linked to gene expression changes across cell types. Dotted and solid lines show *Ppp1r15b*^R658C^ and WT flank height, respectively. (*D*) Schematic of the contextual fear conditioning paradigm. (*E*) Contextual long-term fear memory in mixed sex WT (*n* = 14), *Ppp1r15b*^R658C^*:CamKII-Cre-:Atf4^flox/flox^* (*n* = 20), and *Ppp1r15b*^R658C^*:CamKII-Cre +:Atf4 ^flox/flox^* (*n* = 13) mice (Two-way ANOVA: *F*_2,88_ = 8.78; WT vs. *Ppp1r15b*^R658C^:*CamKII-Cre-:Atf4^flox/flox^, t* = 4.47, *P* < 0.001; *Ppp1r15b*^R658C^*:CamKII-Cre-:Atf4^flox/flox^* vs. *Ppp1r15b*^R658C^*:CamKII-Cre +:Atf4^flox/flox^, t* = 1.55, *P* > 0.99; WT vs. *Ppp1r15b*^R658C^*:CamKII-Cre +:Atf4^flox/flox^, t* = 5.48, *P* < 0.0001). Behavior results were similar when animals were separated by sex. (*F*) Contextual long-term fear memory in mixed sex WT (*n* = 18), *Ppp1r15b*^R658C^*:Gad2-Cre-:Atf4^flox/flox^* (*n* = 13), and *Ppp1r15b*^R658C^*:Gad2-Cre +:Atf4^flox/flox^* (*n* = 17) mice (Two-way ANOVA: *F*_2,90_ = 10.99; WT vs. *Ppp1r15b*^R658C^*:Gad2-Cre-:Atf4^flox/flox^, t* = 3.05, *P* < 0.05; *Ppp1r15b*^R658C^*:Gad2-Cre-:Atf4^flox/flox^* vs. *Ppp1r15b*^R658C^*:Gad2-Cre +:Atf4^flox/flox^, t* = 3.61, *P* < 0.01; WT vs. *Ppp1r15b*^R658C^*:Gad2-Cre +:Atf4^flox/flox^, t* = 7.21, *P* < 0.0001). Behavior results were similar when animals were separated by sex.

To elucidate how the ISR regulates gene expression at the single-cell level, we examined the relationship between chromatin accessibility and transcriptional changes during persistent ISR activation. Using scATAC-seq in WT and *Ppp1r15b*^R658C^ mice, we profiled global chromatin dynamics. We first investigated DNA-binding activity using chromVAR (119), a method that quantifies genome-wide chromatin accessibility at loci containing DNA-binding motifs. The resulting Z-scores reflect deviations from baseline accessibility, providing an estimate of cell type–specific putative TF binding. This analysis revealed striking cell type–specific differences in TF motif enrichment across cell types during persistent ISR activation. Briefly, in *Ppp1r15b*^R658C^ mice, motifs for activator protein 1 (AP-1) components, including FOS and JUN (120), were enriched in glutamatergic neurons, while ATF4 motifs were enriched in GABAergic neurons. In contrast, neither ATF4 nor AP-1 binding motifs were significantly enriched in nonneuronal cell subtypes (Fig. 4*A*). These findings indicate that different cell types reprogram gene expression via distinct downstream effectors upon persistent ISR activation.

To determine whether this selective cell type–specific chromatin remodeling has functional consequences, we utilized Signac (97, 98), a framework for integrating scATAC-seq and gene expression data. Motif analysis of open chromatin regions associated with DEGs confirmed ATF4 enrichment in GABAergic neurons and AP-1 enrichment in glutamatergic neurons (Fig. 4*B* and *SI Appendix*, Fig. S8*C*). Two representative genes illustrate this pattern: the ATF4 target *Gars,* which showed DE and chromatin opening in GABAergic neurons, and *Fbln2*, an AP-1-dependent gene that exhibited DE and chromatin opening in glutamatergic neurons (*SI Appendix*, Fig. S8 *A* and *B*). These examples underscore the cell type–specific gene expression response to persistent ISR activation.

To further dissect TF–DNA interactions, we performed TF footprinting analysis for ATF4 and AP-1 components. This approach estimates TF occupancy by measuring the local protection of DNA motifs from Tn5 transposase integration. When a TF binds its cognate motif, it physically shields the DNA, creating a “footprint” of reduced accessibility at the binding site, while the flanking DNA remains highly accessible due to the displacement of local nucleosomes (121). By averaging these measurements across the entire genome and within individual cell types, one can estimate the regulatory relevance of a specific TF in driving gene expression changes. Enhanced chromatin accessibility surrounding ATF4 motifs was observed in GABAergic neurons, while AP-1 (JUNB) footprints were enriched in glutamatergic neurons (Fig. 4*C*). We further identified selective enrichment of the DNA-binding motifs of JUNB and another AP-1 family member, FOSL2, in glutamatergic neurons (*SI Appendix*, Fig. S8*D*). It is noteworthy that the binding motifs of both JUNB and FOSL2 are distinct from those of other AP-1 components (*SI Appendix*, Fig. S9 *A* and *B*). Similar results were obtained using Pando (122), a multimodal tool that infers gene regulatory networks (GRNs) by integrating scRNA-seq and scATAC-seq data while adjusting for TF expression levels (*SI Appendix*, Fig. S10). These results suggest that persistent ISR activation drives gene expression through cell type–specific TF hierarchies.

The segregation of ATF4 and AP-1 TF activity to GABAergic and glutamatergic neurons, respectively, prompted us to investigate the functional relevance of this divergence. We therefore determined whether conditional deletion of *Atf4* from specific neuronal subtypes differentially affects long-term memory in *Ppp1r15b*^R658C^ mice. To this end, *Ppp1r15b*^R658C^ mice were crossed with *Atf4^flox/flox^*mice, and their progeny were bred with either *Gad2-Ires-Cre* mice [GABAergic-specific; (123)] or *CaMKII-Cre* mice [glutamatergic-specific; (124)] to delete *Atf4* in these neuronal populations.

Long-term memory was assessed using contextual fear conditioning, utilizing freezing behavior 24 h after training as a measure of long-term memory strength (Fig. 4*D*). *Ppp1r15b*^R658C^ mice, characterized by persistent brain ISR activation, displayed impaired contextual long-term memory (Fig. 4*E*), as we recently reported (91). Notably, the deletion of *Atf4* from forebrain glutamatergic neurons did not significantly affect long-term memory in these mice (*Ppp1r15b*^R658C^*:Atf4^flox/flox^:Cre^CaMKII+^* mice, Fig. 4*E*). In contrast, deletion of *Atf4* from GABAergic neurons in *Ppp1r15b*^R658C^ mice (*Ppp1r15b*^R658C^*:Atf4^flox/flox^:Cre^Gad2+^* mice) exacerbated their long-term memory deficits (Fig. 4*F*), indicating that ATF4 has a protective functional role in this neuronal population. These results highlight that ISR downstream effectors play unique roles across different cell types during persistent and maladaptive ISR activation and demonstrate that ATF4-mediated regulation of long-term memory is specific to GABAergic neurons.

## Discussion

Persistent ISR activation in the brain drives long-term memory deficits across a wide array of models of neurological disorders (2). Our single-cell analysis revealed that neurons and glial cells (e.g., astrocytes, oligodendrocytes, and microglia) respond to persistent ISR activation in fundamentally different ways (Fig. 1). We found that GABAergic and glutamatergic populations showed a more pronounced ISR activation than other cell types (Fig. 2*E*), challenging the assumption of a uniform ISR response across all brain cell types and highlighting the complexity of how dysfunctional proteostasis pathways contribute to pathological outcomes in neurological disorders. Mechanistically, ISR kinases phosphorylate eIF2, inhibiting eIF2B and reducing ternary complex formation, thereby suppressing global translation while selectively inducing the expression of ISR-specific genes. Dephosphorylation of eIF2-P by the PPP1R15B•PP1 phosphatase complex suppresses the ISR. Therefore, the relative abundance of ISR components in each cell type likely determines cell-specific outcomes. Although *Ppp1r15b* expression was similar across cell types (*SI Appendix*, Fig. S3), differences in ISR susceptibility likely arise from variation in the levels of methionyl-initiator tRNA (Met-tRNAi), ISR kinases, eIF2, eIF2B, and/or PP1.

Despite prominent ISR-driven gene expression in both major neuronal classes, we observed a functional divergence: *Ppp1r15b*^R658C^ mice exhibited increased GABAergic, but not glutamatergic, synaptic transmission (91). Elevated GABAergic tone impairs long-term memory formation and LTP by disrupting the excitatory-inhibitory balance (125), which is critical for proper cognitive function (126). Supporting this hypothesis, ISR inhibition reduces GABAergic synaptic transmission without affecting glutamatergic synaptic transmission (20, 39). Furthermore, while inhibition of the ISR in other neuronal types, such as GABAergic, glutamatergic, or cholinergic neurons, has been shown to facilitate long-term memory formation (15, 47, 127), we propose that these manipulations converge in promoting synaptic plasticity and restoring the excitatory/inhibitory balance.

Mechanistically, we identified distinct ISR effectors in different neuronal subtypes. In glutamatergic neurons, scATAC-seq revealed that the AP-1 TF JUNB predominates over ATF4. In contrast, ATF4 serves as the principal effector in GABAergic neurons (Fig. 4). Crucially, we found that genetic ATF4 deletion from GABAergic neurons (but not glutamatergic neurons) of *Ppp1r15b*^R658C^ mice further exacerbated their long-term memory deficits. These findings are consistent with a report that lentiviral shRNA-mediated reduction of ATF4 in different brain cells impairs long-term memory (128). However, this contrasts with observations that selective *Atf4* deletion in forebrain excitatory neurons of wild-type mice (127), or the expression of a dominant-negative inhibitor of C/EBP proteins and ATF4 in excitatory forebrain neurons (129), facilitates long-term memory formation. This discrepancy highlights the cell-type- and context-dependent functions of ATF4 and underscores the importance of dissecting ISR mechanisms within specific cell types, as ISR effectors could play distinct roles in health vs. disease states.

Comparatively, our findings distinguish the pathophysiology caused by the *Ppp1r15b*^R658C^ variant from other genetic ISR-associated disorders. For instance, in vanishing white matter disease (VWMD), caused by loss-of-function variant in eIF2B (38), the ISR is primarily activated in astrocytes and oligodendrocytes (38), leading to myelination defects and associated cognitive problems. In contrast, the *Ppp1r15b*^R658C^ variant mainly impacts neurons. Given that neurons depend on precise protein synthesis control regulation for synaptic plasticity and memory (130–133), they are likely the primary mediators of ISR-induced cognitive decline, while glial dysfunction might lead to the white matter pathology seen in VWMD. Indeed, it will be interesting to examine whether neurons are the most susceptible population in other human cognitive disorders (or mouse models) carrying ISR-activating variants, such as those linked to MEHMO (mental deficiency, epilepsy, hypogenitalism, microcephaly, and obesity) syndrome, a genetic ISR-driven, and X-linked disorder (86–88, 134). Together these findings suggest that the pathological consequences of persistent ISR activation depend heavily on which cell types are primarily compromised.

Finally, we leveraged our single-cell data to define a molecular signature of persistent ISR activation. This signaturemaps the landscape of cellular fragility, identifying which cell types are susceptible or resilient to persistent ISR activation. Moreover, the ISR signature is detected in different cell types in the brain of human neurodevelopmental and neurodegenerative disorders (Fig. 3), serving as a broad indicator of cognitive dysfunction and compromised brain health. By providing a metric to assess ISR activation across cell types, tissues, and disease states, this signature offers a valuable resource, enabling the identification of molecular biomarkers associated with disrupted cellular homeostasis.

In summary, our findings provide a fresh perspective on the cellular and molecular mechanisms underlying persistent ISR activation, revealing how cell type–specific vulnerabilities drive the cognitive decline associated with disrupted homeostasis.

## Methods

### Mouse Husbandry.

*Ppp1r15b*^R658C^ mice (91), *Gad2-Ires-Cre* mice [GABAergic-specific; (123)] and *CaMKII-Cre* mice [glutamatergic-specific; (124)] were previously described. ATF4 floxed (*Atf4^flox/flox^*) mice were obtained from Jackson Labs (Bar Harbor, ME). To selectively excise ATF4 in GABAergic or glutamatergic neurons on the *Ppp1r15b*^R658C^ background, *Ppp1r15b*^R658C^ mice were first crossed with the ATF4 floxed line, and the progeny were bred to *Gad2-Ires-Cre* or *CaMKII-Cre* lines, respectively. Behavioral experiments were conducted on a sex-balanced cohort of mice aged 10 to 16 wk. No significant sex-specific differences were observed in the behavioral data. Animals were kept under a 12 h/12 h light/dark cycle (lights on at 7:00 am) and had access to food and water ad libitum. Procedures were approved by Baylor College of Medicine’s and Charles River Laboratories’ Animal Care and Use Committees in accordance with NIH guidelines.

### Nuclei Isolation.

Nuclei were isolated following an established protocol (135) with minor modifications. Frozen cortical tissue (5 to 10 mg) was homogenized in 500 µL of nuclei extraction buffer [0.32 M sucrose, 10 mM Tris pH 7.4, 5 mM CaCl_2_, 3 mM Mg acetate, 1 mM DTT, 0.1 mM EDTA, 0.1% Triton X-100, and 0.2 U/mL Protector RNAse inhibitor (Sigma cat. 3335399001)] using a dounce homogenizer. The was filtered homogenate (70 µm filter) was layered over a 750 µL of sucrose solution (1.8 M sucrose, 10 mM Tris pH 7.4, 3mM Mg acetate, and 1 mM DTT) and centrifuged at 16,000 × g for 30 min at 4 °C. Nuclei were permeabilized following 10× Genomics protocol (CG000375 Rev B; https://www.10xgenomics.com/support/epi-multiome/documentation/steps/sample-prep/nuclei-isolation-from-complex-tissues-for-single-cell-multiome-atac-plus-gene-expression-sequencing) by resuspending in 100 µL lysis buffer (10 mM Tris-HCl pH 7.4, 10 mM NaCl, 3 mM MgCl_2_, 1% BSA, 0.01% Tween 20, 0.01% NP-40, 0.001% digitonin, 1 mM DTT, 1 U/mL of Protector RNAse inhibitor) and incubated 2 min on ice. Nuclei were washed once, resuspended in 30 µL of 1× nuclei buffer with 1 mM DTT and 0.5 U/µL of RNAse inhibitor, then quantified via Countess II FL.

### Single-Nucleus Multiomics.

Transposition, nuclei isolation, barcoding, and library preparation followed the 10X Genomics Chromium Next GEM Single-Cell Multiome protocol (CG000338 Rev F; https://www.10xgenomics.com/support/epi-multiome/documentation/steps/library-prep/chromium-next-gem-single-cell-multiome-atac-plus-gene-expression-reagent-kits-user-guide). Samples (*n* = 14) were processed in one batch, balanced by sex and genotype (*SI Appendix*, Table S1). Each was loaded onto a Chromium Next GEM Chip J to recover ~10,000 nuclei/lane, then sequenced on an Illumina NovaSeq X (Novogene).

### Data Preprocessing.

Cell Ranger ARC (v2.0.2) aligned raw reads to the GRCm38 mouse reference genome. RNA matrices and ATAC fragment were processed using Seurat (136) (v5.2.0) and Signac (97, 98) (v1.14.9), respectively. ATAC reads were requantified across consensus accessible chromatin regions (ACRs; width 20 to 10,000 bp). RNA filtering via PopsicleR (137) (v0.2.1) use thresholds: the maximum number of genes detected per cell (G_RNA_low = 500 and G_RNA_hi = Inf), the range of molecules detected per cell (U_RNA_low = -Inf and U_RNA_hi = 37,000), and the upper limits for mitochondrial, ribosomal, and dissociation gene percentages (percent_mt_hi = 10, percent_ribo_hi = 100, and percent_disso_hi = 100, respectively). ATAC filtering required 500 < nCount_ATAC < 100,000, nucleosome_signal < 3, TSS.enrichment > 1, yielding 85,402 cells. Mean depths per cells were 6,919 unique RNA reads and 10,012 unique ATAC reads, detecting an average of 2,598 genes and 8,602 chromatin peaks per cell. RNA was log-normalized (Seurat’s NormalizeData; (136), scaled (ScaleData) and PCA-reduced (top 2,000 features, 30 PCs). ATAC data underwent latent semantic indexing (LSI, dimensions 2:30) via Signac’s RunTFIDF, FindTopFeatures, and RunSVD (97, 98). Modalities were integrated into a weighted neighborhood graph then projected via Approximation and Projection (UMAP) for visualization.

### Dataset Annotation.

Cell types were identified using a dual approach. First, the MakeAnnotation function within PopsicleR (137) integrated two reference-based tools: SingleR (138) (using bulk mouse RNA-seq) and single-cell Mouse Cell Atlas [scMCA (139)]. Concurrently, Seurat (136) transferred annotations by anchoring query datasets to the Allen Brain Atlas mouse whole-brain Smart-seq v4 dataset ref. 99 via the FindTransferAnchors function [using the first 30 principal components (PCs)]. Results were merged into three annotation levels: Level 1 (GABAergic, glutamatergic, nonneuronal), Level 2 (subclassified nonneuronal), and Level 3 (granular neuronal subtypes).

### Identifying Differentially Expressed Subpopulations.

DE was assessed using MiloDE and SEAcells. Significant genes from both frameworks were ultimately intersected.

- MiloDE (v0.9), built on MiloR (v1.1.0), assigns cells to overlapping neighborhoods on a second-order k-nearest neighbor (kNN) graph (constructed using k = 23, d = 30 in PCA space) for nuanced subpopulation analysis. It performs DE via edge R (covariates: sex and genotype, design matrix: ~sex+geno applying weighted Benjamini–Hochberg correction where *P*-values are weighted by reciprocal neighborhood connectivity. Genes were considered significant if both gene-corrected and neighborhood-corrected *P*-values were <0.05.
- SEAcells (111) aggregates cells into metacells (~75 cells each) via graph-based manifold learning to detect subtle state shifts while preserving heterogeneity. Waypoint cells, selected via minimum-maximum sampling, anchored iterative metacell assignment based on archetypal analysis. The kernel transcriptomic PCA space was initialized with the first 10 principal components. After 50 iterations, reads from WT and *Ppp1r15b*^R658C^ cells were summarized per metacall using soft assignment. Metacell data were normalized, scaled, and PCA-processed in Seurat. DE per Level 3 cell type was calculated using Seurat’s FindMakers (min.pct = 0, logfc.threshold = 0.15,min.cells.feature = 0,min.cells.group = 0).

### Gene Module Identification.

We applied scWGCNA (140)—a single-cell adaptation of WGCNA—to the MiloDE neighborhoods to identify coexpression modules. Topological overlap and adjacency matrices assigned genes to discrete modules, pruning initial memberships lacking correlation with overall module expression. Because module properties depend on the required number of significant neighborhoods, we tested n_hoods_sig.thresh values from 1 to 58 and selected the most representative parameters (n_hoods_sig.thresh = 8, npcs = 10, pval.thresh = 0.1).

This yielded two consistent modules (uniformly increasing and decreasing expression) alongside an oligodendrocyte-specific module (present in <50% of iterations). Intersecting these modules with the significant MiloDE and SEAcells genes produced the final ISR-up (28 genes) and ISR-down (19 genes) signatures. Noncoding, antisense, or genes lacking human orthologs were excluded from the final ISR-down list (*SI Appendix*, Table S2).

### Gene Enrichment Analysis.

Gene enrichment analyses were performed using two tools: the web-based tool, Metascape (141), and GSEA using the package WebGestaltR (DOI: 10.1093/nar/gkz401, v0.4.5). For Metascape, we provided our gene sets as input and all detected genes as the background. Significant Gene Ontology (GO) terms were selected with the threshold of adjusted *P*-value < 0.05.

For each cell type at the L2 level cell annotation, DEGs between WT and *Ppp1r15b*^R658C^ cells (adjusted *P*-value < 0.05) were also analyzed using WebGestaltR. Genes were ranked by their ± (avg FC) X −log10 (adj *P*-value). Enrichment analysis was performed against KEGG pathways and nonredundant Gene Ontology (Biological Process and Molecular Function) databases.

### Validation in External Datasets.

We obtained publicly available datasets for validation, including those related to human Down Syndrome (113) (EGAS00001005691), Parkinson’s disease and Lewy Body Dementia (115) (GSE178265), either through by direct download or from the CZ CELLxGENE CENSUS (142). For validation, we created gene sets of orthologous genes of our up and down gene sets. We then used UCell (143) to calculate signature scores based on the Mann–Whitney *U* statistic. We then performed Mann–Whitney *U* tests between WT and *Ppp1r15b*^R658C^ cells within each L2 level cell annotation using the stat_compare_means function. Our upregulated and downregulated gene sets were validated in mouse samples, and the ISR signature in the human disease datasets. As a final step of validation, we compared our ISR signature to genome wide association studies by calculating a single-cell disease relevance score (scDRS) for each metacell. We employed a one-sided unified Monte Carlo test to evaluate statistical enrichment of each disease, as previously described (65). This method links scRNA-seq data with polygenic disease risk, in a cell type–independent mode. We used the precalculated disease/magma scores for mouse brain data shown exhibited in the example vignette.

### Downstream Single-Cell Epigenetic Analysis.

We identified ACRs within all samples using MACS3 (144) followed by filtering to standard chromosomes and then merging the accessible regions across individual samples. For downstream analyses, we created fragment files based on their SEAcells metacell assignments. We then computed gene activities, and the deviations in accessibility of TFs using the GeneActivity, and RunChromVAR functions in Signac (97, 98). We obtained position weight matrices from the JASPAR2020^83^ database for vertebrates. Motif positioning in open regions of chromatin was determined via the Signac function FindMotifs, and the R package motifmatchr (v.1.24.0). Significant TFs were identified using FindMarkers (adjusted *P*-value < 0.05, test.use = “LR,” latent.vars = “nCount_peaks”). We then linked accessible regions to normalized expression changes (using SCTransform) for all genes using the LinkPeaks Signac command (*P-*value < 0.001 & z-score > 0). To assess TF footprinting in these accessible regions, we used the Footprint function within Signac. Additionally, to explore cell type–specific epigenetic changes linked to the ISR, we created a filtered list of regulatory elements. ACRs were classified as linked to the ISR if they met the following criteria: 1) they were differentially accessible between WT and *Ppp1r15b*^R658C^ cells within each L3 cell annotation (*P*-value < 0.005), 2) they were significantly linked to a gene ± 50 kbps from the TSS, (*P*-value < 0.001 & z-score > 0), and 3) the corresponding gene was differentially expressed between WT and *Ppp1r15b*^R658C^ cells within the same L3 cell annotation (adjusted *P*-value < 0.05). Finally, the results were aggregated on their L1 cell annotation, revealing 134 opening and 57 closing regulatory elements. Hypergeometric tests were performed using the FindMotifs function (*P*-value < 0.05), and peaks were annotated using ChIPseeker (145) (v1.42.0).

### Building GRNs.

We used a GRN inference tool Pando (122) (v1.1.1) to identify TF activity in a cell type–specific manner. Similar to our in-house approach described above, Pando integrates both ATAC and RNA modalities to simultaneously infer regulatory elements for each gene. Pando models expression changes of genes via: 1) selecting linked regulatory genes within ± 10 kbps from the transcription start site (TSS), 2) scanning these regions for TF motifs (for this we used the custom motif point weight matrices provided by Pando), 3) identifying TF-region pairs for each gene, and 4) constructing regression models where the region-TF pairs serves as independent variables and the target genes serve as dependent variables. We applied Pando to our 28 upregulated ISR signature genes, and then to all 163 DEGs that were identified in both MiloDE, and SEAcells. For this analysis, we focused on TFs that were differentially expressed between WT and *Ppp1r15b*^R658C^ cells within L3 cell annotations. We used the find_modules command with the following parameters: p_thresh = 0.1, nvar_thresh = 2, min_genes_per_module = 1, rsq_thresh = 0.05 to identify coregulated modules. Finally, we calculated cell-type activity of a TF by multiplying the mean regulatory coefficient (coef) with the average expression of the TF within each cell type.

### Contextual Long-Term Fear Memory.

Fear conditioning was performed as previously described (20, 39). Mice underwent 3 d of handling (5 min/d) and 2 d of habituation to the conditioning chamber (20 min/d). On the training day, mice were placed in the conditioning chamber for a 2 min (naïve) period, followed by two foot-shocks (0.75 mA, 2 s, 90 s apart). Mice remained in the chamber for 1 min postshock before returning to their home cages. Long-term memory was assessed 24 h later by re-exposing the mice to the conditioning chamber (context) for 5 min. Freezing behavior (defined as immobility excluding respiration) was quantified using real-time video and analyzed by EthoVision XT v17 (Noldus Information Technology BV, Leesburg, VA). Experiments were conducted during the light cycle. Mice were randomized, and investigators were blinded to genotype.

### Statistical Analysis.

Data are presented as mean ± SEM with individual points. Test included the Mann–Whitney *U* test, Spearman correlation analysis, Kruskal–Wallis, EdgeR (MiloDE), Seurat’s FindMarkers (SEACells), hypergeometric tests, and two-way ANOVA with Bonferroni post hoc correction for multiple comparisons. Values (*n*, *t*, *F*, and *P*) are in figure legends. Significance was set at *P* < 0.05 (*P < 0.05, **P < 0.01, ***P < 0.001, and ****P < 0.0001). DE effect sizes are reported as log_2_ fold change. GraphPad Prism (La Jolla, CA) analyzed and plotted behavioral data.

## Supplementary Material

Appendix 01 (PDF)

## Data Availability

The scRNA-seq and scATAC-seq data were deposited in the Gene Expression Omnibus (GEO) database repository (GSE314068) (146). In addition, using the ShinyMultiome.UiO package (https://www.biorxiv.org/content/10.1101/2023.06.20.545756v2) (147), we developed an interactive web application that enables ISR-driven multiomics data visualization, exploration, and integration. This application can be accessed via the following link: https://altoslabs.shinyapps.io/ISR_atlas/ (148). All other data are included in the manuscript and/or *SI Appendix*.

## References

[1] H. P. Harding , An integrated stress response regulates amino acid metabolism and resistance to oxidative stress. Mol. Cell **11**, 619–633 (2003). 12667446 10.1016/s1097-2765(03)00105-9

[2] M. Costa-Mattioli, P. Walter, The integrated stress response: From mechanism to disease. Science **368**, eaat5314 (2020). 32327570 10.1126/science.aat5314 PMC8997189

[3] T. E. Dever, A. C. Dar, F. Sicheri, “The eIF2alpha kinases” in Translational Control in Biology and Medicine, M. B. Mathews, N. Sonenberg, J. W. B. Hershey, Eds. (Cold Spring Harbor Laboratory Press, Cold Spring Harbor, NY, 2007), pp. 319–345.

[4] N. Sonenberg, A. G. Hinnebusch, Regulation of translation initiation in eukaryotes: Mechanisms and biological targets. Cell **136**, 731–745 (2009). 19239892 10.1016/j.cell.2009.01.042 PMC3610329

[5] C. Jousse , Inhibition of a constitutive translation initiation factor 2α phosphatase, CReP, promotes survival of stressed cells. J. Cell Biol. **163**, 767–775 (2003). 14638860 10.1083/jcb.200308075 PMC2173671

[6] I. Novoa, H. Zeng, H. P. Harding, D. Ron, Feedback inhibition of the unfolded protein response by GADD34-mediated dephosphorylation of eIF2alpha. J. Cell Biol. **153**, 1011–1022 (2001). 11381086 10.1083/jcb.153.5.1011 PMC2174339

[7] M. Costa-Mattioli , eIF2α phosphorylation bidirectionally regulates the switch from short- to long-term synaptic plasticity and memory. Cell **129**, 195–206 (2007). 17418795 10.1016/j.cell.2007.01.050 PMC4149214

[8] Z. Jiang , eIF2alpha Phosphorylation-dependent translation in CA1 pyramidal cells impairs hippocampal memory consolidation without affecting general translation. J. Neurosci. **30**, 2582–2594 (2010). 20164343 10.1523/JNEUROSCI.3971-09.2010 PMC2836228

[9] G. V. Di Prisco , Translational control of mGluR-dependent long-term depression and object-place learning by eIF2α. Nat. Neurosci. **17**, 1073–1082 (2014). 24974795 10.1038/nn.3754 PMC4340591

[10] M. A. Trinh , The eIF2alpha kinase PERK limits the expression of hippocampal metabotropic glutamate receptor-dependent long-term depression. Learn. Mem. **21**, 298–304 (2014). 24741110 10.1101/lm.032219.113 PMC3994503

[11] W. Huang , Translational control by eIF2alpha phosphorylation regulates vulnerability to the synaptic and behavioral effects of cocaine. eLife **5**, e12052 (2016). 26928234 10.7554/eLife.12052 PMC4786430

[12] A. N. Placzek , Translational control of nicotine-evoked synaptic potentiation in mice and neuronal responses in human smokers by eIF2alpha. eLife **5** (2016). 10.7554/eLife.12056 PMC4786418 26928076

[13] A. N. Placzek , eIF2alpha-mediated translational control regulates the persistence of cocaine-induced LTP in midbrain dopamine neurons. eLife **5**, e12056 (2016). 27960077 10.7554/eLife.17517 PMC5154759

[14] H. R. Zimmermann , Genetic removal of eIF 2α kinase PERK in mice enables hippocampal L‐ LTP independent of mTORC 1 activity. J. Neurochem. **146**, 133–144 (2018). 29337352 10.1111/jnc.14306 PMC6047941

[15] V. Sharma , eIF2alpha controls memory consolidation via excitatory and somatostatin neurons. Nature **586**, 412–416 (2020). 33029011 10.1038/s41586-020-2805-8 PMC7874887

[16] E. S. Frias , Aberrant cortical spine dynamics after concussive injury are reversed by integrated stress response inhibition. Proc. Natl. Acad. Sci. U.S.A. **119**, e2209427119 (2022). 36227915 10.1073/pnas.2209427119 PMC9586300

[17] N. W. Martinez , PKR-driven ISR signaling controls synaptic translation and structural plasticity in an age-dependent manner. Neurobiol. Dis. **216**, 107113 (2025). 40976378 10.1016/j.nbd.2025.107113

[18] G. Neves, S. F. Cooke, T. V. Bliss, Synaptic plasticity, memory and the hippocampus: A neural network approach to causality. Nat. Rev. Neurosci. **9**, 65–75 (2008). 18094707 10.1038/nrn2303

[19] R. Lamprecht, J. LeDoux, Structural plasticity and memory. Nat. Rev. Neurosci. **5**, 45–54 (2004). 14708003 10.1038/nrn1301

[20] P. J. Zhu , Suppression of PKR promotes network excitability and enhanced cognition by interferon-gamma-mediated disinhibition. Cell **147**, 1384–1396 (2011). 22153080 10.1016/j.cell.2011.11.029 PMC3569515

[21] J. A. Moreno , Sustained translational repression by eIF2alpha-P mediates prion neurodegeneration. Nature **485**, 507–511 (2012). 22622579 10.1038/nature11058 PMC3378208

[22] M. V. Lourenco , TNF-alpha mediates PKR-dependent memory impairment and brain IRS-1 inhibition induced by Alzheimer’s beta-amyloid oligomers in mice and monkeys. Cell Metab. **18**, 831–843 (2013). 24315369 10.1016/j.cmet.2013.11.002

[23] E. Stern, A. Chinnakkaruppan, O. David, N. Sonenberg, K. Rosenblum, Blocking the eIF2α kinase (PKR) enhances positive and negative forms of cortex-dependent taste memory. J. Neurosci. **33**, 2517–2525 (2013). 23392680 10.1523/JNEUROSCI.2322-12.2013 PMC6619168

[24] C. Sidrauski , Pharmacological brake-release of mRNA translation enhances cognitive memory. eLife **2**, e00498 (2013). 23741617 10.7554/eLife.00498 PMC3667625

[25] M. Jian , eIF2alpha dephosphorylation in basolateral amygdala mediates reconsolidation of drug memory. J. Neurosci. **34**, 10010–10021 (2014). 25057203 10.1523/JNEUROSCI.0934-14.2014 PMC6608301

[26] Y. Segev , PKR inhibition rescues memory deficit and ATF4 overexpression in ApoE epsilon4 human replacement mice. J. Neurosci. **35**, 12986–12993 (2015). 26400930 10.1523/JNEUROSCI.5241-14.2015 PMC6605432

[27] F. Mouton-Liger , PKR downregulation prevents neurodegeneration and beta-amyloid production in a thiamine-deficient model. Cell Death Dis. **6**, e1594 (2015). 25590804 10.1038/cddis.2014.552 PMC4669750

[28] G. Batista, J. L. Johnson, E. Dominguez, M. Costa-Mattioli, J. L. Pena, Translational control of auditory imprinting and structural plasticity by eIF2alpha. eLife **5**, e17197 (2016). 28009255 10.7554/eLife.17197 PMC5245967

[29] J. E. Rittiner , Functional genomic analyses of Mendelian and sporadic disease identify impaired eIF2α signaling as a generalizable mechanism for dystonia. Neuron **92**, 1238–1251 (2016). 27939583 10.1016/j.neuron.2016.11.012 PMC5320521

[30] S. M. Eacker , Experience-dependent translational state defined by cell type-specific ribosome profiling. bioRxiv [Preprint] (2017). 10.1101/169425 (Accessed 1 November 2025).

[31] T. Sen, R. Gupta, H. Kaiser, N. Sen, Activation of PERK elicits memory impairment through inactivation of CREB and downregulation of PSD95 after traumatic brain injury. J. Neurosci. **37**, 5900–5911 (2017). 28522733 10.1523/JNEUROSCI.2343-16.2017 PMC5473207

[32] A. Chou , Inhibition of the integrated stress response reverses cognitive deficits after traumatic brain injury. Proc. Natl. Acad. Sci. U.S.A. **114**, E6420–E6426 (2017). 28696288 10.1073/pnas.1707661114 PMC5547647

[33] K. D. Hwang, M. S. Bak, S. J. Kim, S. Rhee, Y. S. Lee, Restoring synaptic plasticity and memory in mouse models of Alzheimer’s disease by PKR inhibition. Mol. Brain **10**, 57 (2017). 29233183 10.1186/s13041-017-0338-3 PMC5727890

[34] E. Chesnokova, N. Bal, P. Kolosov, Kinases of eIF2a switch translation of mRNA subset during neuronal plasticity. Int. J. Mol. Sci. **18**, 2213 (2017). 29065505 10.3390/ijms18102213 PMC5666893

[35] V. Sharma , Local inhibition of PERK enhances memory and reverses age-related deterioration of cognitive and neuronal properties. J. Neurosci. **38**, 648–658 (2018). 29196323 10.1523/JNEUROSCI.0628-17.2017 PMC6596193

[36] C. T. Werner, M. T. Stefanik, M. Milovanovic, A. Caccamise, M. E. Wolf, Protein translation in the nucleus accumbens is dysregulated during cocaine withdrawal and required for expression of incubation of cocaine craving. J. Neurosci. **38**, 2683–2697 (2018). 29431650 10.1523/JNEUROSCI.2412-17.2018 PMC5852654

[37] P. A. Melas , Cannabinoid modulation of eukaryotic initiation factors (eIF2α and eIF2B1) and behavioral cross-sensitization to cocaine in adolescent rats. Cell Rep. **22**, 2909–2923 (2018). 29539420 10.1016/j.celrep.2018.02.065

[38] Y. L. Wong , eIF2B activator prevents neurological defects caused by a chronic integrated stress response. eLife **8**, e42940 (2019). 30624206 10.7554/eLife.42940 PMC6326728

[39] P. J. Zhu , Activation of the ISR mediates the behavioral and neurophysiological abnormalities in Down syndrome. Science **366**, 843–849 (2019). 31727829 10.1126/science.aaw5185 PMC7299149

[40] M. Tible , PKR knockout in the 5xFAD model of Alzheimer’s disease reveals beneficial effects on spatial memory and brain lesions. Aging Cell **18**, e12887 (2019). 30821420 10.1111/acel.12887 PMC6516179

[41] P. Shrestha , Cell-type-specific drug-inducible protein synthesis inhibition demonstrates that memory consolidation requires rapid neuronal translation. Nat. Neurosci. **23**, 281–292 (2020). 31959934 10.1038/s41593-019-0568-z PMC7147976

[42] R. Moradi Majd, M. Mayeli, F. Rahmani, Pathogenesis and promising therapeutics of Alzheimer disease through eIF2α pathway and correspondent kinases. Metab. Brain Dis. **35**, 1241–1250 (2020). 32681467 10.1007/s11011-020-00600-8

[43] P. Shrestha , Amygdala inhibitory neurons as loci for translation in emotional memories. Nature **586**, 407–411 (2020). 33029009 10.1038/s41586-020-2793-8 PMC7572709

[44] S. Bond, C. Lopez-Lloreda, P. J. Gannon, C. Akay-Espinoza, K. L. Jordan-Sciutto, The integrated stress response and phosphorylated eukaryotic initiation factor 2alpha in neurodegeneration. J. Neuropathol. Exp. Neurol. **79**, 123–143 (2020). 31913484 10.1093/jnen/nlz129 PMC6970450

[45] K. Krukowski , Small molecule cognitive enhancer reverses age-related memory decline in mice. eLife **9**, e62048 (2020). 33258451 10.7554/eLife.62048 PMC7721440

[46] N. W. Martinez, F. E. Gomez, S. Matus, The potential role of protein kinase R as a regulator of age-related neurodegeneration. Front. Aging Neurosci. **13**, 638208 (2021). 33994991 10.3389/fnagi.2021.638208 PMC8113420

[47] A. R. Helseth , Cholinergic neurons constitutively engage the ISR for dopamine modulation and skill learning in mice. Science **372**, eabe1931 (2021). 33888613 10.1126/science.abe1931 PMC8457366

[48] M. M. Oliveira , Correction of eIF2-dependent defects in brain protein synthesis, synaptic plasticity, and memory in mouse models of Alzheimer’s disease. Sci. Signal. **14**, eabc5429 (2021). 33531382 10.1126/scisignal.abc5429 PMC8317334

[49] M. Lopez-Grancha , A novel selective PKR inhibitor restores cognitive deficits and neurodegeneration in Alzheimer disease experimental models. J. Pharmacol. Exp. Ther. **378**, 262–275 (2021). 34531308 10.1124/jpet.121.000590

[50] L. Jiang , Inhibition of the integrated stress response reverses oxidative stress damage-induced postoperative cognitive dysfunction. Front. Cell. Neurosci. **16**, 992869 (2022). 36212697 10.3389/fncel.2022.992869 PMC9534309

[51] Z. Hu , Inhibition of the ISR abrogates mGluR5-dependent long-term depression and spatial memory deficits in a rat model of Alzheimer’s disease. Transl. Psychiatry **12**, 96 (2022). 35260557 10.1038/s41398-022-01862-9 PMC8904583

[52] A. B. Kawa , Positive allosteric modulation of mGlu(1) reverses cocaine-induced behavioral and synaptic plasticity through the integrated stress response and oligophrenin-1. Biol. Psychiatry **92**, 871–879 (2022). 35871097 10.1016/j.biopsych.2022.05.008 PMC10656746

[53] I. Carreras, Y. Jung, J. Lopez-Benitez, C. M. Tognoni, A. Dedeoglu, Fingolimod mitigates memory loss in a mouse model of Gulf War Illness amid decreasing the activation of microglia, protein kinase R, and NFkappaB. Neurotoxicology **96**, 197–206 (2023). 37160207 10.1016/j.neuro.2023.05.006 PMC10334821

[54] V. Sharma , mRNA translation in astrocytes controls hippocampal long-term synaptic plasticity and memory. Proc. Natl. Acad. Sci. U.S.A. **120**, e2308671120 (2023). 38015848 10.1073/pnas.2308671120 PMC10710058

[55] N. Dhir , PERK inhibitor, GSK2606414, ameliorates neuropathological damage, memory and motor functional impairments in cerebral ischemia via PERK/p-eIF2a/ATF4/CHOP signaling. Metab. Brain Dis. **38**, 1177–1192 (2023). 36847967 10.1007/s11011-023-01183-w

[56] M. Hayakawa-Ogura, Tana, T. Nakagawa, M. Itoh, GADD34 suppresses eIF2alpha phosphorylation and improves cognitive function in Alzheimer’s disease-model mice. Biochem. Biophys. Res. Commun. **654**, 112–119 (2023). 36907138 10.1016/j.bbrc.2023.02.077

[57] M. M. Oliveira , The integrated stress response effector GADD34 is repurposed by neurons to promote stimulus-induced translation. Cell Rep. **43**, 113670 (2024). 38219147 10.1016/j.celrep.2023.113670 PMC10964249

[58] P. Goswami , Downregulation of ATF-4 attenuates the endoplasmic reticulum stress-mediated neuroinflammation and cognitive impairment in experimentally induced Alzheimer’s disease model. Mol. Neurobiol. **61**, 5071–5082 (2024). 38159199 10.1007/s12035-023-03861-3

[59] W. Jia , ISRIB ameliorates spatial learning and memory impairment induced by adolescent intermittent ethanol exposure in adult male rats. Neurochem. Int. **179**, 105834 (2024). 39142353 10.1016/j.neuint.2024.105834

[60] S. Y. Wang , Inhibition of the integrated stress response prevents natural forgetting and corrects accelerated forgetting associated with epilepsy. Mol. Neurobiol. **62**, 6059–6069 (2025). 39708234 10.1007/s12035-024-04669-5

[61] X. Feng , Circular RNA aptamers targeting neuroinflammation ameliorate Alzheimer disease phenotypes in mouse models. Nat. Biotechnol. **44**, 454–463 (2025). 40164764 10.1038/s41587-025-02624-w

[62] A. P. Kalinin, E. S. Zubkova, M. Y. Menshikov, Y. V. Parfyonova, ISR modulators in neurological diseases. Curr. Neuropharmacol. **23**, 1184–1214 (2025). 39995125 10.2174/011570159X361653250213114821 PMC12308010

[63] K. Lin , Chronic integrated stress response causes dysregulated cholesterol synthesis in white matter disease. JCI Insight **10**, e188459 (2025). 40674686 10.1172/jci.insight.188459 PMC12406721

[64] M. Liang , PKR inhibitor C16 regulates HIV-gp120 induced neuronal injury and cognitive impairment in vivo and in vitro models. Neurochem. Res. **50**, 70 (2025). 39752056 10.1007/s11064-024-04322-6

[65] Y. Chen , Integrated stress response inhibition prolongs the lifespan of a Pelizaeus-Merzbacher disease mouse model by increasing oligodendrocyte survival. Nat. Commun. **17**, 1285 (2025). 41453885 10.1038/s41467-025-68045-0 PMC12868624

[66] D. Ho-Tieng, V. Sharma, N. Sonenberg, C. G. Gkogkas, A. Khoutorsky, The integrated stress response in the brain: Cell type-specific functions in health and neurological disorders. Trends Neurosci. **48**, 808–821 (2025). 40903367 10.1016/j.tins.2025.08.002

[67] M. M. Oliveira , Neuron type-specific mRNA translation programs provide a gateway for memory consolidation. bioRxiv [Preprint] (2025). 10.1101/2025.01.13.632784 (Accessed 1 November 2025).

[68] B. N. Flores , Investigational eIF2B activator DNL343 modulates the integrated stress response in preclinical models of TDP-43 pathology and individuals with ALS in a randomized clinical trial. Nat. Commun. **16**, 7690 (2025). 40825784 10.1038/s41467-025-63031-y PMC12361401

[69] N. P. Ilyin , Neurotranscriptomic and behavioral effects of ISRIB, and its therapeutic effects in the traumatic brain injury model in zebrafish. Brain Res. **1848**, 149329 (2025). 39537125 10.1016/j.brainres.2024.149329

[70] K. Chen , Selective removal of astrocytic PERK protects against glymphatic impairment and decreases toxic aggregation of beta-amyloid and tau. Neuron **113**, 2438–2454.e2436 (2025). 40403715 10.1016/j.neuron.2025.04.027 PMC12210236

[71] J. Khan , Arsenic trioxide underpins delayed neuroinflammation and impaired synaptic integrity involving integrative stress response signaling. bioRxiv [Preprint] (2025). 10.64898/2025.11.30.691457 (Accessed 1 November 2025).

[72] H. Sun , Restoring glucose metabolism in Alzheimer’s disease by targeting integrated stress response. Neurotherapeutics **22**, e00618 (2025). 40480904 10.1016/j.neurot.2025.e00618 PMC12491788

[73] T. Zhao , Targeting the integrated stress response with ISRIB enhances CREB/BDNF signaling and attenuates cognitive deficits in a rat model of vascular cognitive impairment. Eur. J. Pharmacol. **1011**, 178457 (2026). 41380823 10.1016/j.ejphar.2025.178457

[74] D. Yan, Y. Jiao, X. Zhang, H. Yan, PERK inhibition mitigates acrylamide-induced tau phosphorylation and synaptic deficits via the GSK-3beta and ATF4 pathways in human neuroblastoma SH-SY5Y cells. Ecotoxicol. Environ. Saf. **309**, 119583 (2026). 41406896 10.1016/j.ecoenv.2025.119583

[75] S. Y. Liu, Y. Zuo, X. Jin, X. Kuang, S. W. Tian, Integrated stress response inhibition rescues inflammation-associated accelerated forgetting of recognition memory in mice. Psychopharmacology, 10.1007/s00213-026-07046-3 (2026). 41870529

[76] N. Mahmood , The ISR downstream effector ATF4 promotes mGluR-dependent long-term depression and associated behavior. Proc. Natl. Acad. Sci. U.S.A. **123**, e2532796123 (2026). 41706892 10.1073/pnas.2532796123 PMC12933080

[77] M. Morel, J. Couturier, C. Lafay-Chebassier, M. Paccalin, G. Page, PKR, the double stranded RNA-dependent protein kinase as a critical target in Alzheimer’s disease. J. Cell Mol. Med. **13**, 1476–1488 (2009). 19602051 10.1111/j.1582-4934.2009.00849.x PMC3828860

[78] D. Hrabos , Unfolded protein response markers Grp78 and eIF2alpha are upregulated with increasing alpha-synuclein levels in Lewy body disease. Neuropathol. Appl. Neurobiol. **50**, e12999 (2024). 39036837 10.1111/nan.12999

[79] J. J. Hoozemans , Activation of the unfolded protein response in Parkinson’s disease. Biochem. Biophys. Res. Commun. **354**, 707–711 (2007). 17254549 10.1016/j.bbrc.2007.01.043

[80] A. R. Isaac , Defective regulation of the eIF2-eIF2B translational axis underlies depressive-like behavior in mice and correlates with major depressive disorder in humans. Transl. Psychiatry **14**, 397 (2024). 39349438 10.1038/s41398-024-03128-y PMC11442801

[81] A. Fogli, O. Boespflug-Tanguy, The large spectrum of eIF2B-related diseases. Biochem. Soc. Trans. **34**, 22–29 (2006). 16246171 10.1042/BST20060022

[82] S. Thuppanattumadam Ananthasubramanian, G. Arunachal, H. Padmanabha, R. R. Mahale, Adult-onset EIF2B-pathies: A clinical, imaging and genetic profiling with literature review. Can. J. Neurol. Sci., 10.1017/cjn.2024.308 (2024). 39450483

[83] B. Abdulkarim , A missense mutation in PPP1R15B causes a syndrome including diabetes, short stature, and microcephaly. Diabetes **64**, 3951–3962 (2015). 26159176 10.2337/db15-0477 PMC4713904

[84] K. D. Kernohan , Homozygous mutation in the eukaryotic translation initiation factor 2alpha phosphatase gene, PPP1R15B, is associated with severe microcephaly, short stature and intellectual disability. Hum. Mol. Genet. **24**, 6293–6300 (2015). 26307080 10.1093/hmg/ddv337 PMC4614701

[85] A. Fatalska , Recruitment of trimeric eIF2 by phosphatase non-catalytic subunit PPP1R15B. Mol. Cell **84**, 506–521.e511 (2024). 38159565 10.1016/j.molcel.2023.12.011 PMC7615683

[86] G. Borck , eIF2γ mutation that disrupts eIF2 complex integrity links intellectual disability to impaired translation initiation. Mol. Cell **48**, 641–646 (2012). 23063529 10.1016/j.molcel.2012.09.005 PMC3513554

[87] M. Skopkova , EIF2S3 mutations associated with severe X-linked intellectual disability syndrome MEHMO. Hum. Mutat. **38**, 409–425 (2017). 28055140 10.1002/humu.23170 PMC6267786

[88] S. Moortgat , Two novel EIF2S3 mutations associated with syndromic intellectual disability with severe microcephaly, growth retardation, and epilepsy. Am. J. Med. Genet. A **170**, 2927–2933 (2016). 27333055 10.1002/ajmg.a.37792

[89] G. Park , Neurodegeneration risk factor, EIF2AK3 (PERK), influences tau protein aggregation. J. Biol. Chem. **299**, 102821 (2023). 36563857 10.1016/j.jbc.2022.102821 PMC9852698

[90] Y. Liu, M. Boone, P. Walter, M. Costa-Mattioli, “The integrated stress response: From basic mechanisms to a unified approach to treating cognitive disorders of different etiologies” in Learning and Memory: A Comprehensive Reference, J. T. Wixted, Ed. (Elsevier, ed. 3, 2025), pp. 476–491.

[91] L. C. Reineke , Harnessing viral strategies to reverse cognitive dysfunction through the integrated stress response. Science **392**, eaea8782 (2026). 41926581 10.1126/science.aea8782

[92] R. J. Siarey , Altered signaling pathways underlying abnormal hippocampal synaptic plasticity in the Ts65Dn mouse model of Down syndrome. J. Neurochem. **98**, 1266–1277 (2006). 16895585 10.1111/j.1471-4159.2006.03971.x

[93] R. S. Vartak, A. Rodin, S. Oddo, Differential activation of the mTOR/autophagy pathway predicts cognitive performance in APP/PS1 mice. Neurobiol. Aging **83**, 105–113 (2019). 31585361 10.1016/j.neurobiolaging.2019.08.018

[94] S. Mulherkar , RhoA-ROCK inhibition reverses synaptic remodeling and motor and cognitive deficits caused by traumatic brain injury. Sci. Rep. **7**, 10689 (2017). 28878396 10.1038/s41598-017-11113-3 PMC5587534

[95] A. Zeisel , Molecular architecture of the mouse nervous system. Cell **174**, 999–1014.e1022 (2018). 30096314 10.1016/j.cell.2018.06.021 PMC6086934

[96] B. Tasic , Adult mouse cortical cell taxonomy revealed by single cell transcriptomics. Nat. Neurosci. **19**, 335–346 (2016). 26727548 10.1038/nn.4216 PMC4985242

[97] T. Stuart, A. Srivastava, S. Madad, C. A. Lareau, R. Satija, Author correction: Single-cell chromatin state analysis with Signac. Nat. Methods **19**, 257 (2022). 10.1038/s41592-022-01393-7 34997233

[98] T. Stuart, A. Srivastava, S. Madad, C. A. Lareau, R. Satija, Single-cell chromatin state analysis with Signac. Nat. Methods **18**, 1333–1341 (2021). 34725479 10.1038/s41592-021-01282-5 PMC9255697

[99] Z. Yao , A taxonomy of transcriptomic cell types across the isocortex and hippocampal formation. Cell **184**, 3222–3241.e3226 (2021). 34004146 10.1016/j.cell.2021.04.021 PMC8195859

[100] M. T. Buckley , Cell-type-specific aging clocks to quantify aging and rejuvenation in neurogenic regions of the brain. Nat. Aging **3**, 121–137 (2023). 37118510 10.1038/s43587-022-00335-4 PMC10154228

[101] K. M. Vattem, R. C. Wek, Reinitiation involving upstream ORFs regulates ATF4 mRNA translation in mammalian cells. Proc. Natl. Acad. Sci. U.S.A. **101**, 11269–11274 (2004). 15277680 10.1073/pnas.0400541101 PMC509193

[102] P. D. Lu, H. P. Harding, D. Ron, Translation reinitiation at alternative open reading frames regulates gene expression in an integrated stress response. J. Cell Biol. **167**, 27–33 (2004). 15479734 10.1083/jcb.200408003 PMC2172506

[103] H. P. Harding , Regulated translation initiation controls stress-induced gene expression in mammalian cells. Mol. Cell **6**, 1099–1108 (2000). 11106749 10.1016/s1097-2765(00)00108-8

[104] S. Dey , Both transcriptional regulation and translational control of ATF4 are central to the integrated stress response. J. Biol. Chem. **285**, 33165–33174 (2010). 20732869 10.1074/jbc.M110.167213 PMC2963398

[105] I. Ben-Sahra, G. Hoxhaj, S. J. H. Ricoult, J. M. Asara, B. D. Manning, mTORC1 induces purine synthesis through control of the mitochondrial tetrahydrofolate cycle. Science **351**, 728–733 (2016). 26912861 10.1126/science.aad0489 PMC4786372

[106] M. E. Torrence , The mTORC1-mediated activation of ATF4 promotes protein and glutathione synthesis downstream of growth signals. eLife **10**, e63326 (2021). 33646118 10.7554/eLife.63326 PMC7997658

[107] Y. Park, A. Reyna-Neyra, L. Philippe, C. C. Thoreen, mTORC1 balances cellular amino acid supply with demand for protein synthesis through post-transcriptional control of ATF4. Cell Rep. **19**, 1083–1090 (2017). 28494858 10.1016/j.celrep.2017.04.042 PMC5811220

[108] I. M. N. Wortel, L. T. van der Meer, M. S. Kilberg, F. N. van Leeuwen, Surviving stress: Modulation of ATF4-mediated stress responses in normal and malignant cells. Trends Endocrinol. Metab. **28**, 794–806 (2017). 28797581 10.1016/j.tem.2017.07.003 PMC5951684

[109] Y. Baran , MetaCell: Analysis of single-cell RNA-seq data using K-nn graph partitions. Genome Biol. **20**, 206 (2019). 31604482 10.1186/s13059-019-1812-2 PMC6790056

[110] A. Missarova, E. Dann, L. Rosen, R. Satija, J. Marioni, Leveraging neighborhood representations of single-cell data to achieve sensitive DE testing with miloDE. Genome Biol. **25**, 189 (2024). 39026254 10.1186/s13059-024-03334-3 PMC11256449

[111] S. Persad , SEACells infers transcriptional and epigenomic cellular states from single-cell genomics data. Nat. Biotechnol. **41**, 1746–1757 (2023). 36973557 10.1038/s41587-023-01716-9 PMC10713451

[112] J. Han , ER-stress-induced transcriptional regulation increases protein synthesis leading to cell death. Nat. Cell Biol. **15**, 481–490 (2013). 23624402 10.1038/ncb2738 PMC3692270

[113] C. R. Palmer, C. S. Liu, W. J. Romanow, M. H. Lee, J. Chun, Altered cell and RNA isoform diversity in aging Down syndrome brains. Proc. Natl. Acad. Sci. U.S.A. **118** (2021). 10.1073/pnas.2114326118 PMC8617492 34795060

[114] M. Dierssen, Down syndrome: The brain in trisomic mode. Nat. Rev. Neurosci. **13**, 844–858 (2012). 23165261 10.1038/nrn3314

[115] T. Kamath , Single-cell genomic profiling of human dopamine neurons identifies a population that selectively degenerates in Parkinson’s disease. Nat. Neurosci. **25**, 588–595 (2022). 35513515 10.1038/s41593-022-01061-1 PMC9076534

[116] M. J. Zhang , Polygenic enrichment distinguishes disease associations of individual cells in single-cell RNA-seq data. Nat. Genet. **54**, 1572–1580 (2022). 36050550 10.1038/s41588-022-01167-z PMC9891382

[117] L. Devi, M. Ohno, Phospho-eIF2α level is important for determining abilities of BACE1 reduction to rescue cholinergic neurodegeneration and memory defects in 5XFAD mice. PLoS One **5**, e12974 (2010). 20886088 10.1371/journal.pone.0012974 PMC2944882

[118] T. Ma , Suppression of eIF2α kinases alleviates Alzheimer’s disease–related plasticity and memory deficits. Nat. Neurosci. **16**, 1299–1305 (2013). 23933749 10.1038/nn.3486 PMC3756900

[119] A. N. Schep, B. Wu, J. D. Buenrostro, W. J. Greenleaf, chromVAR: Inferring transcription-factor-associated accessibility from single-cell epigenomic data. Nat. Methods **14**, 975–978 (2017). 28825706 10.1038/nmeth.4401 PMC5623146

[120] M. Karin, Z. Liu, E. Zandi, AP-1 function and regulation. Curr. Opin. Cell Biol. **9**, 240–246 (1997). 9069263 10.1016/s0955-0674(97)80068-3

[121] M. Bentsen , ATAC-seq footprinting unravels kinetics of transcription factor binding during zygotic genome activation. Nat. Commun. **11**, 4267 (2020). 32848148 10.1038/s41467-020-18035-1 PMC7449963

[122] J. S. Fleck , Inferring and perturbing cell fate regulomes in human brain organoids. Nature **621**, 365–372 (2023). 36198796 10.1038/s41586-022-05279-8 PMC10499607

[123] H. Taniguchi , A resource of Cre driver lines for genetic targeting of GABAergic neurons in cerebral cortex. Neuron **71**, 995–1013 (2011). 21943598 10.1016/j.neuron.2011.07.026 PMC3779648

[124] I. Dragatsis, S. Zeitlin, CaMKIIalpha-Cre transgene expression and recombination patterns in the mouse brain. Genesis **26**, 133–135 (2000). 10686608 10.1002/(sici)1526-968x(200002)26:2<133::aid-gene10>3.0.co;2-v

[125] P. D. Whissell , Acutely increasing deltaGABA(A) receptor activity impairs memory and inhibits synaptic plasticity in the hippocampus. Front. Neural Circuits **7**, 146 (2013). 24062648 10.3389/fncir.2013.00146 PMC3775149

[126] R. C. Froemke, Plasticity of cortical excitatory-inhibitory balance. Annu. Rev. Neurosci. **38**, 195–219 (2015). 25897875 10.1146/annurev-neuro-071714-034002 PMC4652600

[127] N. Mahmood , The ISR downstream target ATF4 represses long-term memory in a cell type-specific manner. Proc. Natl. Acad. Sci. U.S.A. **121**, e2407472121 (2024). 39047038 10.1073/pnas.2407472121 PMC11295034

[128] S. Pasini, C. Corona, J. Liu, L. A. Greene, M. L. Shelanski, Specific downregulation of hippocampal ATF4 reveals a necessary role in synaptic plasticity and memory. Cell Rep. **11**, 183–191 (2015). 25865882 10.1016/j.celrep.2015.03.025 PMC4822418

[129] A. Chen , Inducible enhancement of memory storage and synaptic plasticity in transgenic mice expressing an inhibitor of ATF4 (CREB-2) and C/EBP proteins. Neuron **39**, 655–669 (2003). 12925279 10.1016/s0896-6273(03)00501-4

[130] M. Costa-Mattioli, W. S. Sossin, E. Klann, N. Sonenberg, Translational control of long-lasting synaptic plasticity and memory. Neuron **61**, 10–26 (2009). 19146809 10.1016/j.neuron.2008.10.055 PMC5154738

[131] E. Klann, T. E. Dever, Biochemical mechanisms for translational regulation in synaptic plasticity. Nat. Rev. Neurosci. **5**, 931–942 (2004). 15550948 10.1038/nrn1557

[132] R. J. Kelleher III, A. Govindarajan, S. Tonegawa, Translational regulatory mechanisms in persistent forms of synaptic plasticity. Neuron **44**, 59–73 (2004). 15450160 10.1016/j.neuron.2004.09.013

[133] M. Costa-Mattioli, N. Sonenberg, J. D. Richter, “Translational regulatory mechanisms in synaptic plasticity and memory storage” in Translational Control in Health and Disease, J. W. Hershey, Ed. (Elsevier, Taramani, 2009), **vol. 90**, pp. 293–311. 10.1016/S1877-1173(09)90008-4 20374745

[134] S. K. Young-Baird , Suppression of MEHMO syndrome mutation in eIF2 by small molecule ISRIB. Mol. Cell **77**, 875–886.e877 (2020). 31836389 10.1016/j.molcel.2019.11.008 PMC7035991

[135] A. G. Anderson , Single nucleus multiomics identifies ZEB1 and MAFB as candidate regulators of Alzheimer’s disease-specific cis-regulatory elements. Cell Genom. **3**, 100263 (2023). 36950385 10.1016/j.xgen.2023.100263 PMC10025452

[136] Y. Hao , Dictionary learning for integrative, multimodal and scalable single-cell analysis. Nat. Biotechnol. **42**, 293–304 (2024). 37231261 10.1038/s41587-023-01767-y PMC10928517

[137] F. Grandi , popsicleR: A R package for pre-processing and quality control analysis of single cell RNA-seq data. J. Mol. Biol. **434**, 167560 (2022). 35662457 10.1016/j.jmb.2022.167560

[138] D. Aran , Reference-based analysis of lung single-cell sequencing reveals a transitional profibrotic macrophage. Nat. Immunol. **20**, 163–172 (2019). 30643263 10.1038/s41590-018-0276-y PMC6340744

[139] H. Sun, Y. Zhou, L. Fei, H. Chen, G. Guo, scMCA: A tool to define mouse cell types based on single-cell digital expression. Methods Mol. Biol. **1935**, 91–96 (2019). 30758821 10.1007/978-1-4939-9057-3_6

[140] C. Feregrino, P. Tschopp, Assessing evolutionary and developmental transcriptome dynamics in homologous cell types. Dev. Dyn. **251**, 1472–1489 (2022). 34114716 10.1002/dvdy.384 PMC9545966

[141] Y. Zhou , Metascape provides a biologist-oriented resource for the analysis of systems-level datasets. Nat. Commun. **10**, 1523 (2019). 30944313 10.1038/s41467-019-09234-6 PMC6447622

[142] S. Abdulla ; CZI Cell Science Program, CZ CELLxGENE discover: A single-cell data platform for scalable exploration, analysis and modeling of aggregated data. Nucleic Acids Res. **53**, D886–D900 (2025). 39607691 10.1093/nar/gkae1142 PMC11701654

[143] M. Andreatta, S. J. Carmona, UCell: Robust and scalable single-cell gene signature scoring. Comput. Struct. Biotechnol. J. **19**, 3796–3798 (2021). 34285779 10.1016/j.csbj.2021.06.043 PMC8271111

[144] Y. Zhang , Model-based analysis of ChIP-Seq (MACS). Genome Biol. **9**, R137 (2008). 18798982 10.1186/gb-2008-9-9-r137 PMC2592715

[145] G. Yu, L. G. Wang, Q. Y. He, ChIPseeker: An R/Bioconductor package for ChIP peak annotation, comparison and visualization. Bioinformatics **31**, 2382–2383 (2015). 25765347 10.1093/bioinformatics/btv145

[146] K. Torkenczy , Data from “Distinct cell type-specific mechanisms underlie cognitive dysfunction during persistent integrated stress response activation.” Gene Expression Omnibus. https://www.ncbi.nlm.nih.gov/geo/query/acc.cgi?acc=GSE314068. Deposited 17 December 2025. 10.1073/pnas.2537017123 PMC13389509 42446983

[147] A. Akshay , ShinyMultiome.UiO: An interactive open-source framework utilizing Seurat Objects for visualizing single-cell Multiomes. bioRxiv [Preprint] (2023). 10.1101/2023.06.20.545756 (Accessed 1 November 2026).

[148] K. Torkenczy , ISR atlas. Altos Labs. https://altoslabs.shinyapps.io/ISR_atlas/. Accessed 1 November 2026.

