# Supplementary material for: Distinct cell type–specific mechanisms underlie cognitive dysfunction during persistent integrated stress response activation
Source: Proc Natl Acad Sci U S A. 2026 Jul 14;123(29):e2537017123. doi: 10.1073/pnas.2537017123 (PMC13389509; doi:10.1073/pnas.2537017123)
Supplement: Supplementary file 1 — Appendix 01 (PDF) [file pnas.2537017123.sapp.pdf]

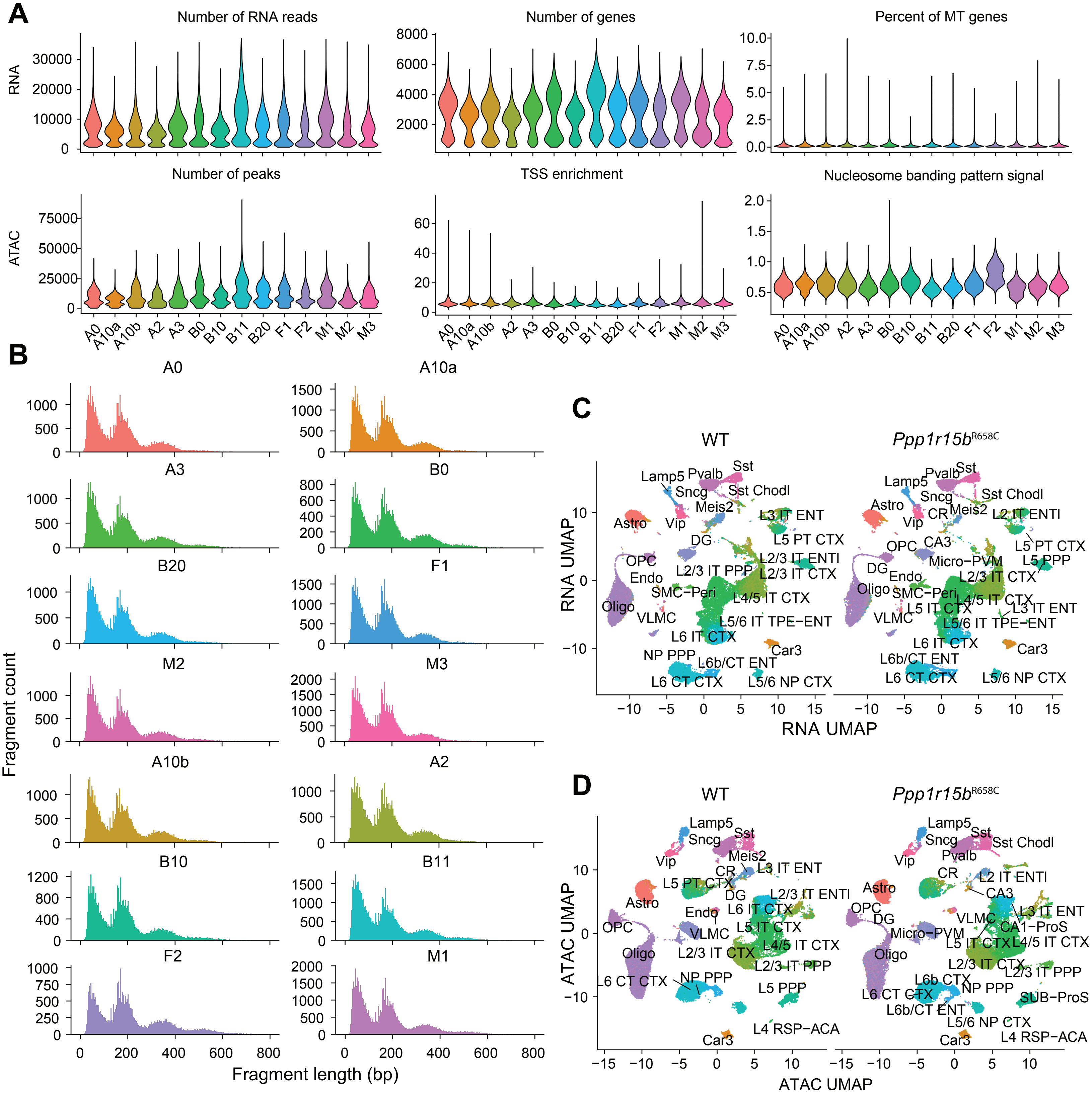

Fig. S1. Torkenczy *et al.*

Figure S1. Quality control of single-cell sequencing.

(A) Violin plots showing RNA (top panels) and scATAC quality metrics (bottom panels). From left to right: RNA metrics include the number of unique reads per cell, the number of detected genes per cell, and percentage of mitochondrial genes detected per cell. scATAC metrics include the number of peaks detected per cell, the enrichment of Transcription Start Sites (TSS), and the nucleosome banding pattern (Kruskal-Wallis *H* test n = 102,269: RNA reads χ^2 (13) = 5493.12, $P < 0.0001$, η^2 [*H*] = 0.0536, Number of genes χ^2 (13) = 5701.24, $P < 0.0001$, η^2 [*H*] = 0.0556, Percent of MT genes χ^2 (13) = 4362.00, $P < 0.0001$, η^2 [*H*] = 0.0425, Number of peaks χ^2 (13) = 4517.08, $P < 0.0001$, η^2 [*H*] = 0.0440, TSS enrichment χ^2 (13) = 4594.63, $P < 0.0001$, η^2 [*H*] = 0.0448, Nucleosome banding pattern signal χ^2 (13) = 13391.91, $P < 0.0001$, η^2 [*H*] = 0.1310).

(B) Fragment count distribution broken down by fragment length for each sample.

(C) UMAP of scRNA-seq data with I3 cell annotations.

(D) UMAP of scATAC-seq data with I3 cell annotations.

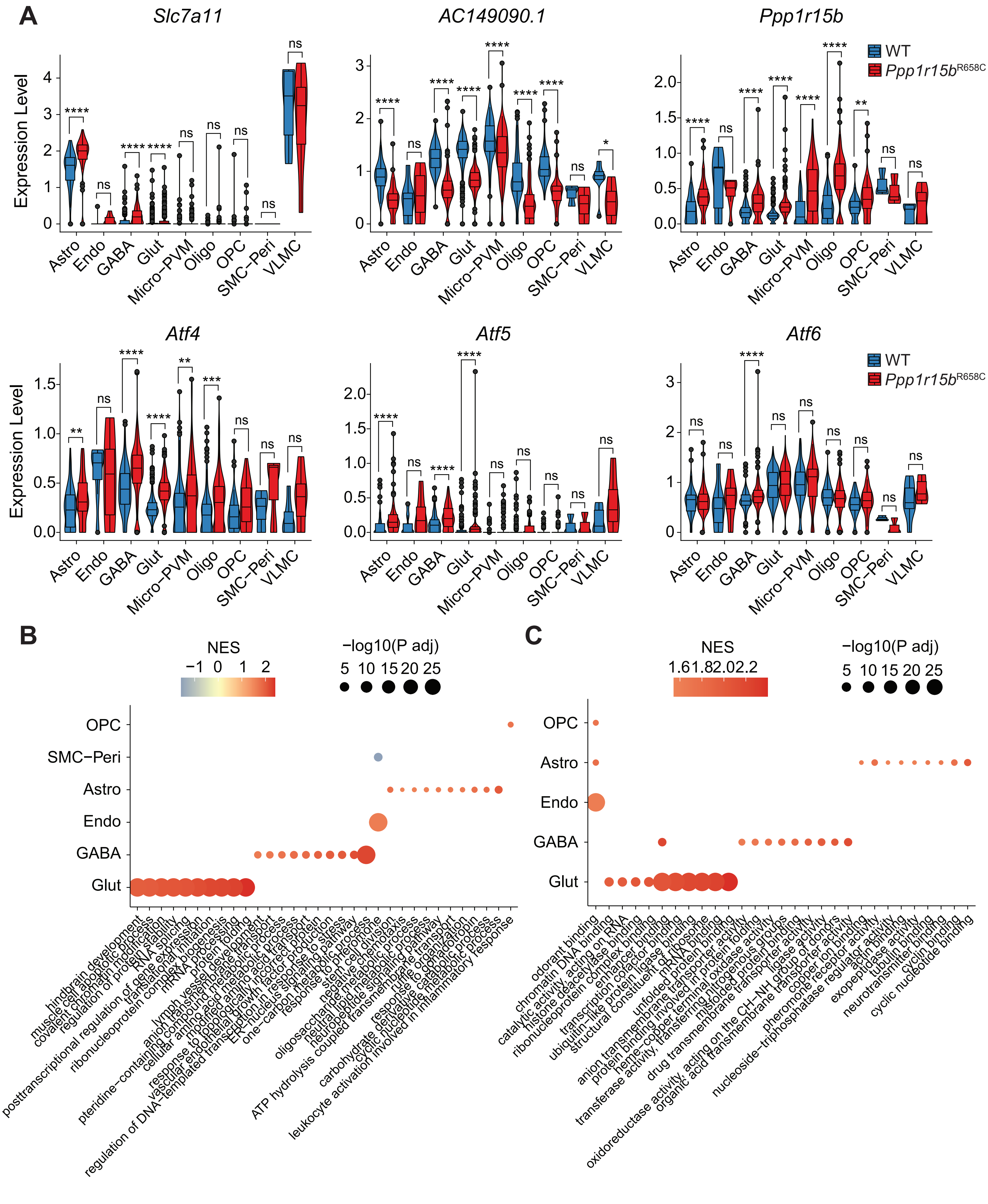

Fig. S2. Torkenczy *et al.*

Figure S2. Representative differentially expressed genes across cell types and enrichment analysis of molecular function and biological process.

(A) Violin plots showing differentially expressed genes (DEGs) between WT and *Ppp1r15b*^{R658C} cell populations, stratified by cell type. The Mann-Whitney *U* test was used to assess significant increases in ISR genes, with significance indicated as follows: ns is $P > 0.05$, $*P < 0.05$, $**P < 0.01$, $***P < 0.001$, $****P < 0.0001$.

(B) Dot plots of molecular function gene set enrichment analysis (GSEA) for DEGs between WT and *Ppp1r15b*^{R658C}, broken down by cell type. Significant terms were selected with an adjusted *P*-value threshold of < 0.05. Dot color represents the normalized enrichment score (NES, effect size), while dot size reflects the -log10 of the adjusted *P*-value.

(C) Dot plots of biological process gene set enrichment analysis (GSEA) for DEGs between WT and *Ppp1r15b*^{R658C}, broken down by cell type. Significant terms were selected with an adjusted *P*-value threshold of < 0.05. Dot color represents the normalized enrichment score (NES, effect size), and dot size represents the -log10 of the adjusted *P*-value.

A

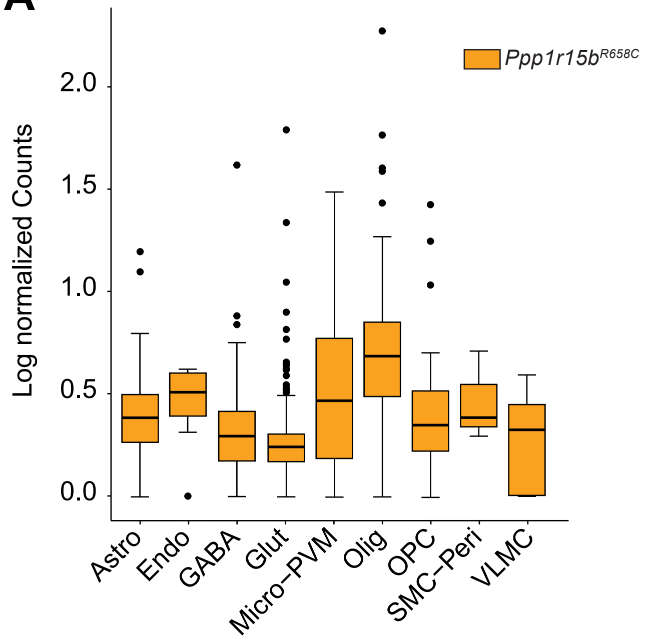

Fig. S3. *Torkenczy et al.*

Figure S3. *Ppp1r15b* levels are consistent across cell lineages and do not drive cell type-specific transcriptional responses.

Box plot of expression levels of *Ppp1r15b* across cell types (Kruskal-Wallis *H* test n = 1,109: χ^2 (8) = 216.26, $P < 2.2 \times 10^{-16}$). The results revealed a statistically significant, though minor, variation in *Ppp1r15b* transcript levels between the groups, which did not correlate with *Atf4* or ATF4-target gene (*Ddit3* and *Asns*) expression when a Spearman's rank correlation analysis was performed (*Atf4*, ρ = 0.01, $P > 0.05$; *Ddit3*, ρ = 0.05, $P > 0.05$; *Asns*, ρ = -0.03, $P > 0.05$).

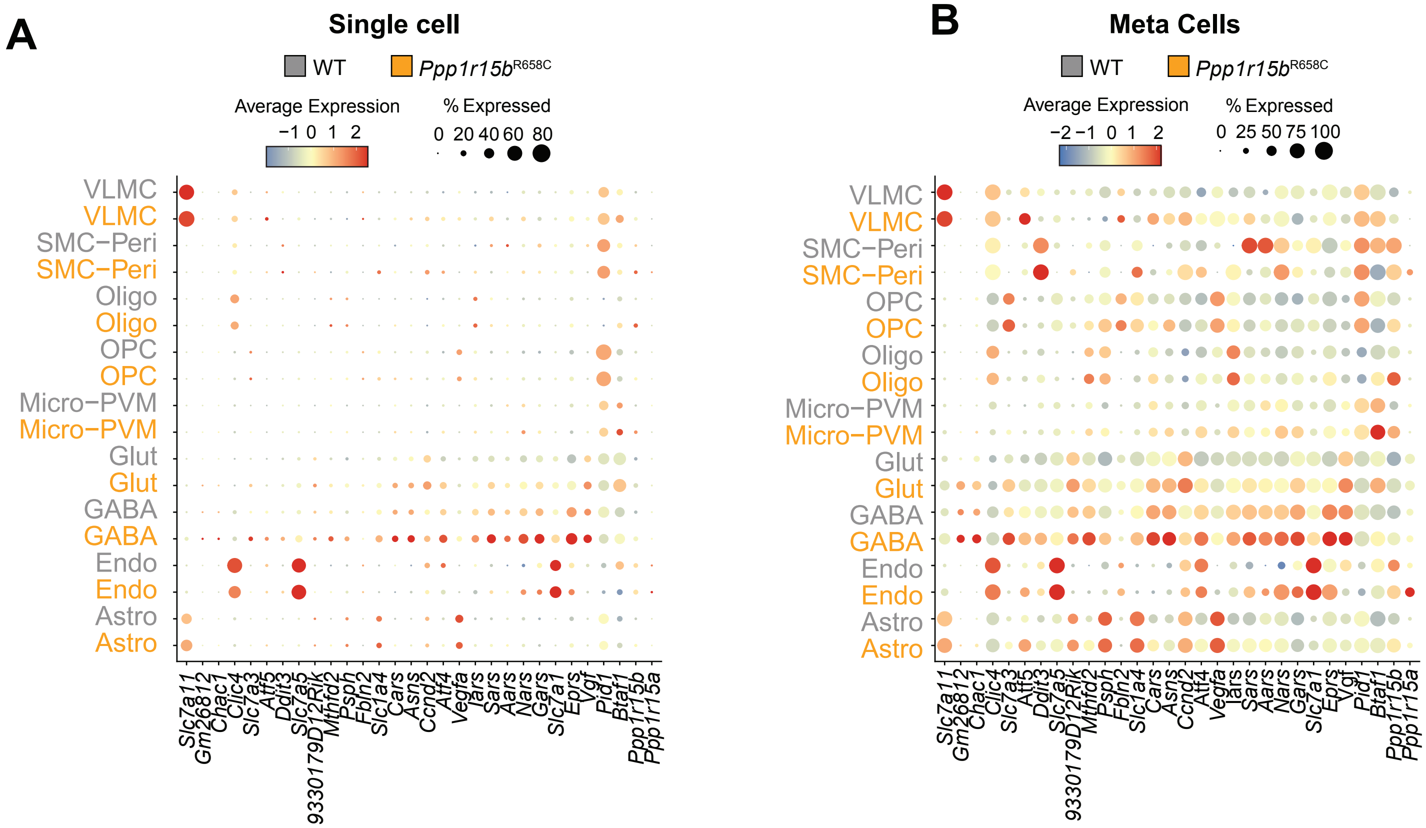

Fig. S4. Torkenczy *et al.*

Figure S4. Comparison of standard single-cell analysis and cluster-free analysis of the ISR upregulated gene set.

(A) Dot plot showing expression of the ISR-specific upregulated gene set across L3 cell types using single-cell normalized expression. The percent of cells expressing each gene is indicated by the dot size.

(B) Dot plot showing expression of the ISR-specific upregulated gene set across L3 cell types using a metacell-based aggregation of normalized expression. The percent of metacells expressing each gene is indicated by the dot size.

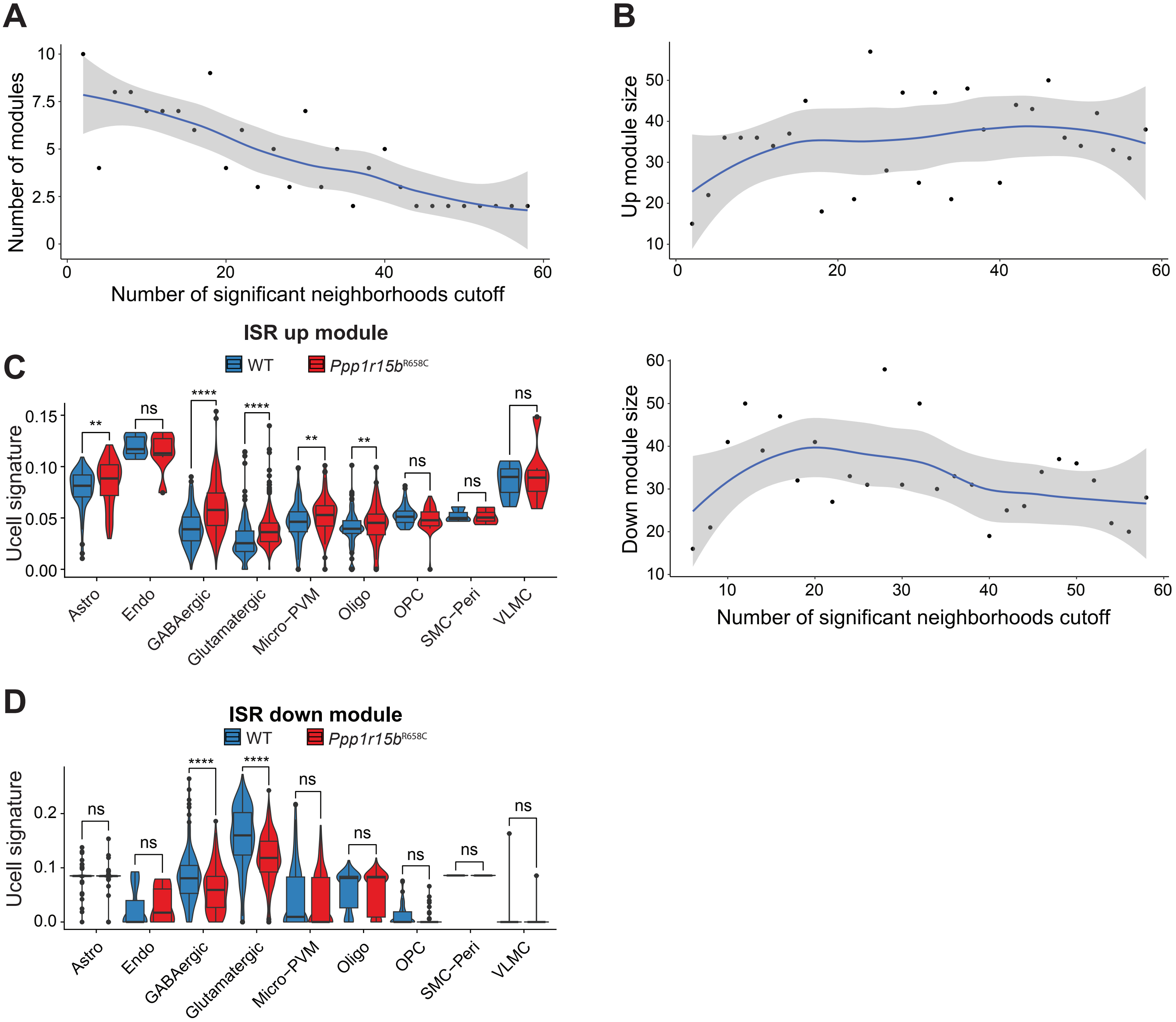

Fig. S5. Torkenczy *et al.*

Figure S5. Neighborhood analyses of ISR-related changes in the transcriptome.

(A) Iterative analysis of the number of significant neighborhoods required for a gene to be included in Weighted Gene Network Analysis (WGCNA). The number of modules identified in each iteration is shown on the y-axis.

(B) Number of genes included in ISR upregulated gene set (top) and ISR downregulated gene set (bottom) as a function of the increasing significant cutoff for neighborhood inclusion.

(C) U-cell enrichment scores of identified ISR upregulated gene set across cell types. We used the Mann-Whitney *U* test for statistics with significance indicated (**$P < 0.01$, ****$P < 0.0001$).

(D) U-cell enrichment scores of ISR downregulated gene set across cell types. We used the Mann-Whitney *U* test for statistics with significance indicated (****$P < 0.0001$).

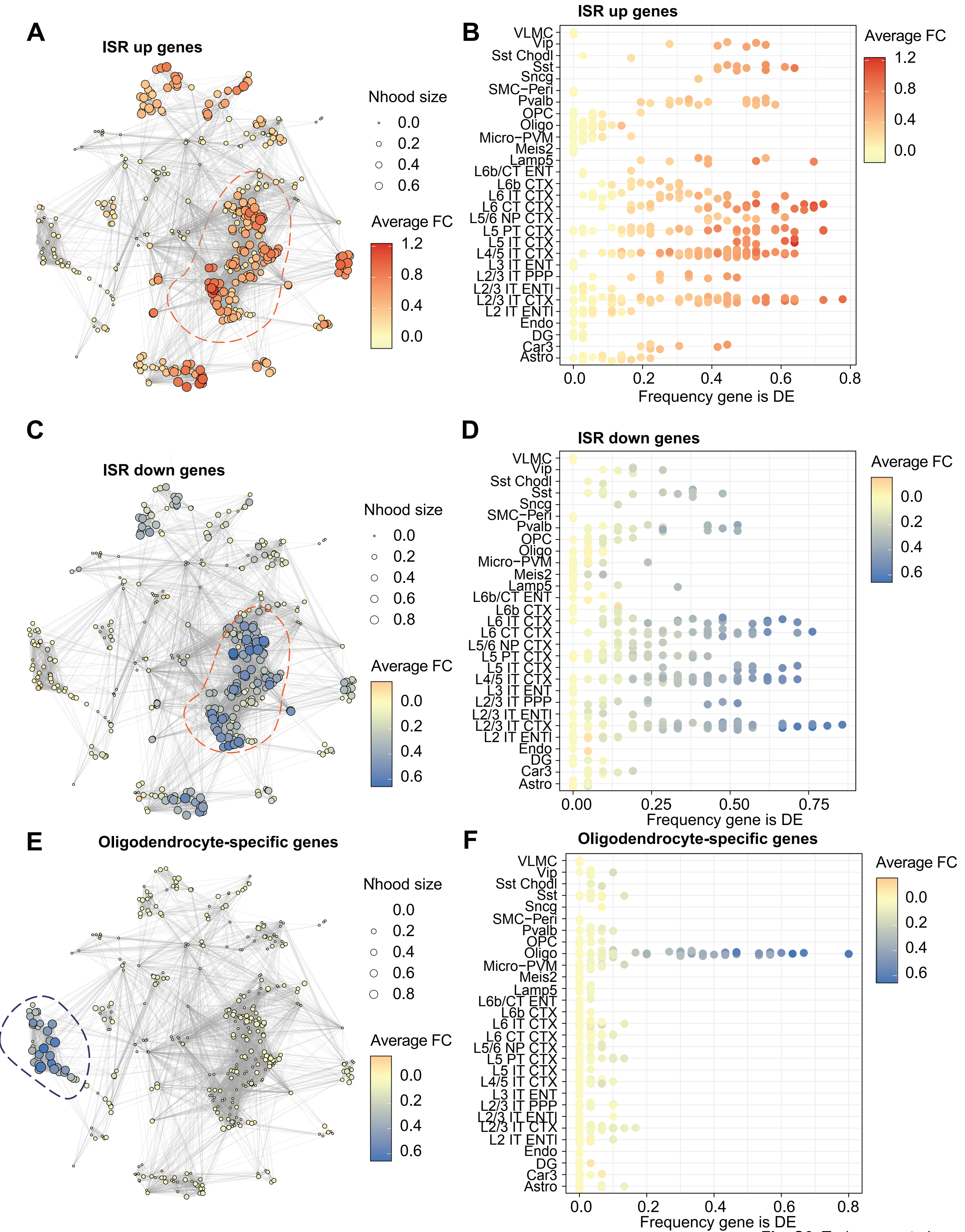

Fig. S6. Torkenczy *et al.*

Figure S6. ISR gene set neighborhoods in MiloDE and enrichment across different cell types and brain regions.

(A) ISR upregulated gene set neighborhoods identified using MiloDE, with average fold-change between $Ppp1r15b^{R658C}$ and WT cells per neighborhood. Positive fold-change (effect-size) values indicate upregulation of the module in $Ppp1r15b^{R658C}$ cells, while negative values indicate downregulation. Neighborhood size is represented by node size, and overlaps between neighborhoods are shown by edges.

(B) ISR upregulated gene set enrichment across l3 cell type annotations.

(C) ISR downregulated gene set neighborhoods from MiloDE, with average fold-change between $Ppp1r15b^{R658C}$ and WT cells per neighborhood. Positive fold-change (effect-size) values indicate up-regulation in $Ppp1r15b^{R658C}$ cells, while negative values indicate down-regulation. Neighborhood size is represented by node size, and overlaps between neighborhoods are shown by connecting edges.

(D) ISR downregulated gene set enrichment across l3 cell type annotations.

(E) Oligodendrocyte-specific gene set from MiloDE, with average fold-change between $Ppp1r15b^{R658C}$ and WT cells per neighborhood. Positive fold-change (effect-size) values indicate upregulation in $Ppp1r15b^{R658C}$ cells, while negative values indicate downregulation. Neighborhood size is reflected in node size, and overlaps between neighborhoods are shown by edges.

(F) Oligodendrocyte-specific gene set enrichment across l3 cell type annotations.

Figure S7. Expression patterns of the ISR upregulated module in external datasets.

(A) Dot plot showing the expression of the ISR upregulated gene set across brain cell types in Down Syndrome patients. The average log fold change (effect-size) is represented by dot color, and the -log10 adjusted *P*-value is indicated by dot size.

(B) Dot plot showing the expression of the ISR upregulated gene set across brain cell types in Parkinson's Disease patients. The average log fold change (effect-size) is represented by dot color, and the -log10 adjusted *P*-value is indicated by dot size.

(C) Dot plot showing the expression of the ISR upregulated gene set across brain cell types in Lewy body dementia patients. The average log fold change (effect-size) is represented by dot color, and the -log10 adjusted *P*-value is indicated by dot size.

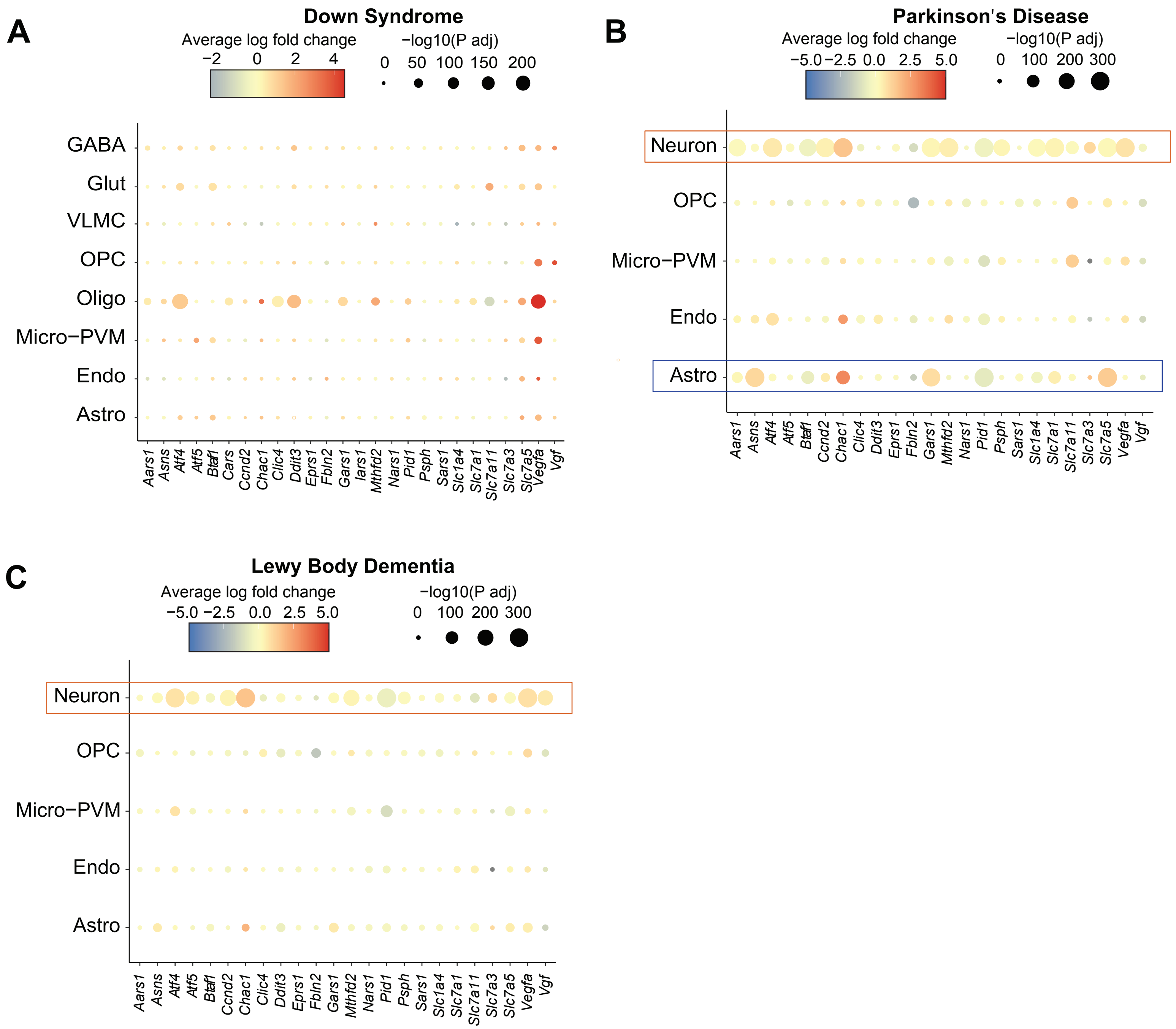

Fig. S7. Torkenczy *et al.*

Figure S8. Epigenetic regulation of cell type-specific ISR modulation.

(A) Linked scATAC peaks regulating *Gars* expression (left) and mRNA expression (right) across brain cell types, demonstrating differential accessibility and expression in GABAergic neurons. ±20 kb from the transcription start site (TSS) is shown. Differential accessibility is indicated by grey lines.

(B) Linked scATAC peaks regulating *Fbln2* expression (left) and mRNA expression (right) across brain cell types, demonstrating differential accessibility and expression in glutamatergic neurons. ±20 kb from the TSS is shown. Differential accessibility is indicated by grey lines.

(C) Volcano plots showing enrichment of transcription factor motifs in linked regulatory regions for GABAergic (left), glutamatergic (middle), and non-neuronal (right) cell types. A hypergeometric test was performed using GC-content matched background peaks (*P*-value < 0.001 and z-score > 0).

(D) Bias-corrected scATAC-seq footprinting signal centered around FOSL2 motifs, aggregated from peaks linked to gene expression changes across cell types. Dotted horizontal lines represent the flank height for $Ppp1r15b^{R658C}$ and WT for comparison.

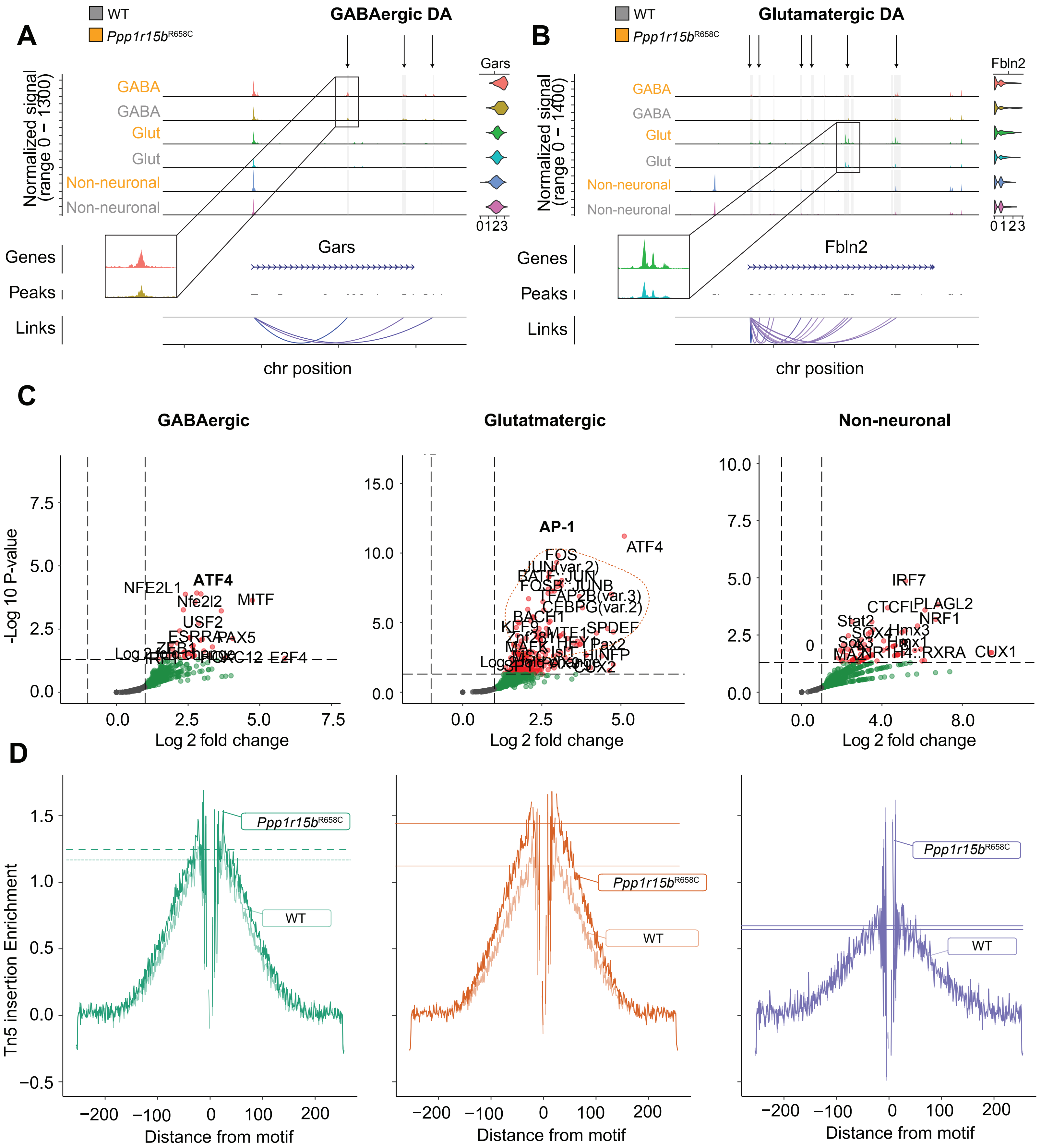

Fig. S8. Torkenczy *et al.*

Figure S9. Comparison of DNA binding motifs across the AP1 transcription factor family.

(A) Jaccard similarity of accessibility of regions regulated by each transcription factor within the AP1 family of transcription factors.

(B) Jaccard similarity of genes regulated by each transcription factor within the AP1 family of transcription factors.

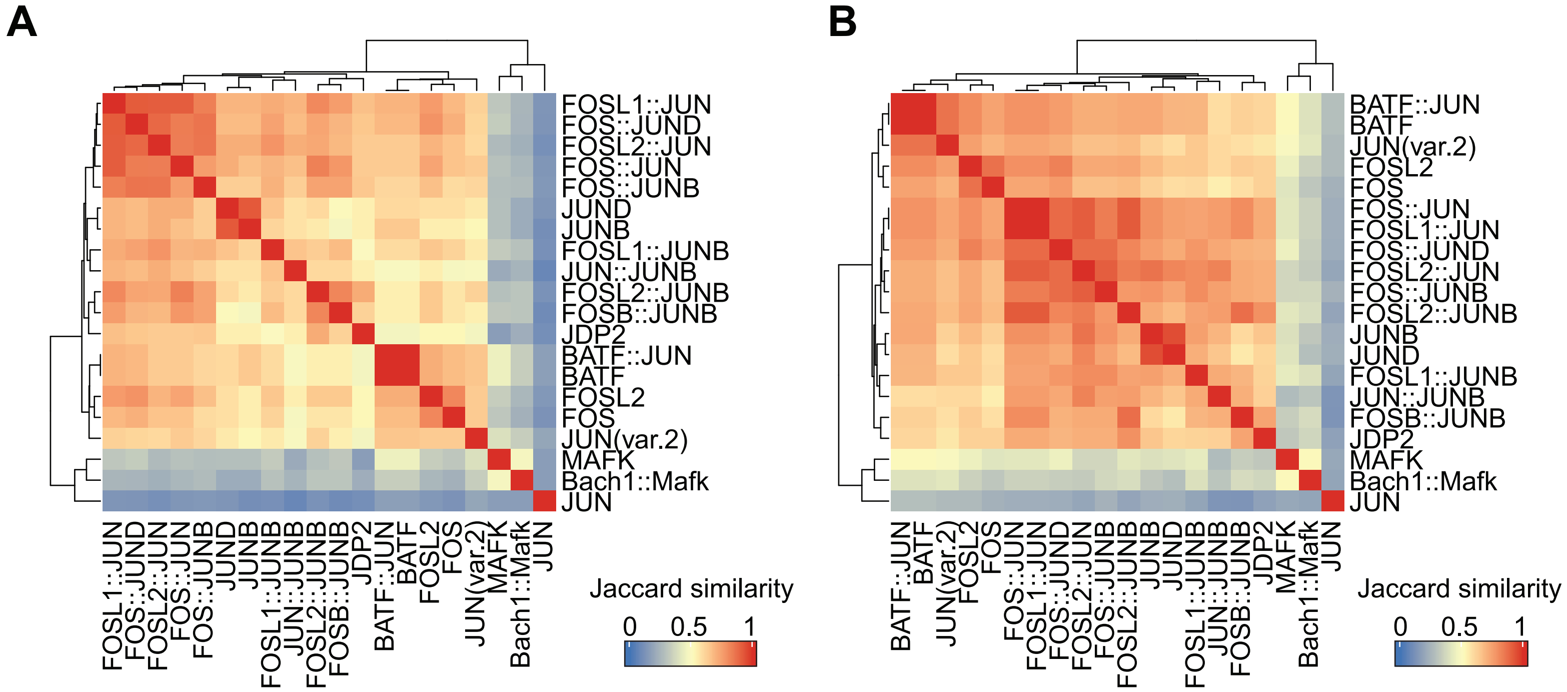

Fig. S9. Torkenczy *et al.*

Figure S10. Pando analysis supports a role for ATF4 and other transcription factors in the persistent ISR signature.

Hierarchy of regulation centered around ATF4 using Pando gene regulatory networks.

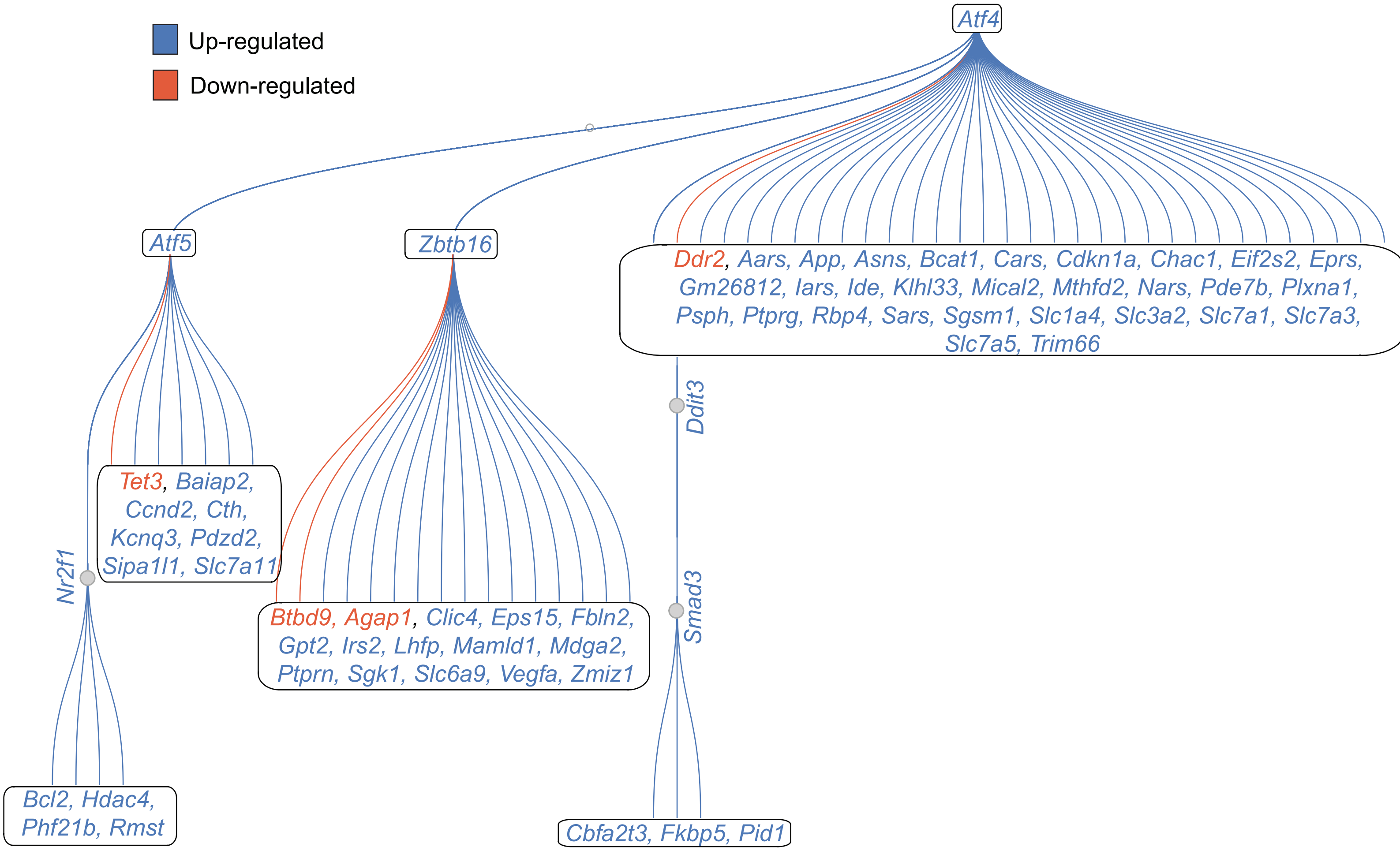

Fig. S10. Torkenczy *et al.*

Table S1: Meta data for animals used in multiomics.

Sample Identifier	Genotype	Sex	Nuclei Count
**A10a** (Sample 2)	*Ppp1r15b*^{R658C}	Female	8,153
**A2** (Sample 4)	*Ppp1r15b*^{R658C}	Female	7,972
**B10** (Sample 7)	*Ppp1r15b*^{R658C}	Female	6,259
**A0** (Sample 1)	*Ppp1r15b*^{R658C}	Male	6,323
**A10b** (Sample 3)	*Ppp1r15b*^{R658C}	Male	5,644
**A3** (Sample 5)	*Ppp1r15b*^{R658C}	Male	7,416
**B0** (Sample 6)	*Ppp1r15b*^{R658C}	Male	2,797
**B11** (Sample 8)	WT	Female	4,103
**B20** (Sample 9)	WT	Female	4,438
**F1** (Sample 10)	WT	Female	5,386
**F2** (Sample 11)	WT	Female	4,097
**M1** (Sample 12)	WT	Male	6,599
**M2** (Sample 13)	WT	Male	7,405
**M3** (Sample 14)	WT	Male	8,810
**Total**			**85,402**

Table S2: Genes excluded from ISR down signature.

Gene ID	Description
Bc1	Rodent-specific Brain specific RNA 1
Gm15680	Noncoding / predicted (lncRNA, antisense, or low-confidence transcript) with no known human ortholog*
Gm16226	Noncoding / predicted (lncRNA, antisense, or low-confidence transcript) with no known human ortholog*
Gm16351	Noncoding / predicted (lncRNA, antisense, or low-confidence transcript) with no known human ortholog*
Gm20275	Noncoding / predicted (lncRNA, antisense, or low-confidence transcript) with no known human ortholog*
Gm26749	Noncoding / predicted (lncRNA, antisense, or low-confidence transcript) with no known human ortholog*
Gm32014	Noncoding / predicted (lncRNA, antisense, or low-confidence transcript) with no known human ortholog*
Gm37240	Noncoding / predicted (lncRNA, antisense, or low-confidence transcript) with no known human ortholog*
Gm48091	Noncoding / predicted (lncRNA, antisense, or low-confidence transcript) with no known human ortholog*
Gm48239	Noncoding / predicted (lncRNA, antisense, or low-confidence transcript) with no known human ortholog*
Gm20642	Noncoding / predicted (lncRNA, antisense, or low-confidence transcript) with no known human ortholog*
Gm41609	Noncoding / predicted (lncRNA, antisense, or low-confidence transcript) with no known human ortholog*
Gm46102	Noncoding / predicted (lncRNA, antisense, or low-confidence transcript) with no known human ortholog*
Gm11149	Noncoding / predicted (lncRNA, antisense, or low-confidence transcript) with possible human ortholog*
Gm47271	Noncoding / predicted (lncRNA, antisense, or low-confidence transcript) with no known human ortholog*
Gm1992	Noncoding / predicted (lncRNA, antisense, or low-confidence transcript) with no known human ortholog*
Gm19951	Noncoding / predicted (lncRNA, antisense, or low-confidence transcript) with no known human ortholog*
Gm42303	Noncoding / predicted (lncRNA, antisense, or low-confidence transcript) with possible human ortholog*
9630028H03Rik	Noncoding / predicted (lncRNA, antisense, or low-confidence transcript) with no known human ortholog*
1700024B18Rik	Noncoding / predicted (lncRNA, antisense, or low-confidence transcript) with no known human ortholog*
4930587E11Rik	Noncoding / predicted (lncRNA, antisense, or low-confidence transcript) with no known human ortholog*
A230004M16Rik	Noncoding / predicted (lncRNA, antisense, or low-confidence transcript) with no known human ortholog*

D130009I18Rik	Noncoding / predicted (lncRNA, antisense, or low-confidence transcript) with no known human ortholog*
AC149090.1	Mouse ortholog of human *PISD* (phosphatidylserine decarboxylase)*
Nrg3os	Opposite strand anti-sense non-coding RNA, no known human ortholog*
Pcsk2os2	Opposite strand anti-sense non-coding RNA, no known human ortholog*
Sorbs2os	Opposite strand anti-sense non-coding RNA, no known human ortholog*

*Based on Ensembl/GENCODE/NCBI annotations

Table S3: Spearman's correlation analysis of *Ppp1r15b* levels versus ISR signature genes

Gene Pair (Ppp1r15b vs.)	Spearman ρ	p-value	Interpretation
Atf4	0.01	0.7359	No Correlation (Uncoupled)
Ddit3 (Chop)	0.05	0.0925	No Correlation (Uncoupled)
Atf5	-0.03	0.3206	No Correlation (Uncoupled)
Asns	-0.03	0.3167	No Correlation (Uncoupled)
Slc7a11	-0.04	0.1572	No Correlation (Uncoupled)
Gars	-0.04	0.2228	No Correlation (Uncoupled)
Eprs	0	0.938	No Correlation (Uncoupled)
Sars	-0.01	0.7346	No Correlation (Uncoupled)
Aars	0.03	0.3897	No Correlation (Uncoupled)
Nars	0.02	0.4856	No Correlation (Uncoupled)
Cars	-0.03	0.3753	No Correlation (Uncoupled)
Btaf1	-0.02	0.4113	No Correlation (Uncoupled)
Pid1	-0.05	0.0984	No Correlation (Uncoupled)
Chac1	-0.08	0.0068	Negligible / Inverse
Slc1a4	-0.07	0.0126	Negligible / Inverse
Slc7a5	-0.09	0.0037	Negligible / Inverse
Slc7a3	-0.1	0.0011	Negligible / Inverse
Gm26812	-0.14	< 0.0001	Weak Inverse
Ccnd2	-0.16	< 0.0001	Weak Inverse
Slc7a1	-0.16	< 0.0001	Weak Inverse
Vgf	-0.17	< 0.0001	Weak Inverse
Fbln2	-0.18	< 0.0001	Weak Inverse
9330179D12Rik	-0.18	< 0.0001	Weak Inverse
Vegfa	0.11	0.0003	Weak Positive
Mthfd2	0.14	< 0.0001	Weak Positive
Psph	0.21	< 0.0001	Weak Positive
Iars	0.23	< 0.0001	Weak Positive
Clic4	0.29	< 0.0001	Weak Positive
Atf4 vs. Asns (Control)	0.42	< 0.0001	Strongly Coupled
Atf4 vs. Chac1 (Control)	0.34	< 0.0001	Strongly Coupled
